# Supplementary material for: Context dependent prediction in DNA sequence using neural networks
Source: PeerJ. 2022 Sep 20;10:e13666. doi: 10.7717/peerj.13666 (PMC9504454; doi:10.7717/peerj.13666)
Supplement: Supplemental Information 1 [file peerj-10-13666-s001.pdf]

Supplementary A: Context dependent prediction in DNA sequence using neural networks. Methods.

Christian Grønbæk, Yuhu Liang, Desmond Elliott, and Anders Krogh

April 30, 2022

Contents

Neural networks **SA2**
Generalities . . . SA2
Architectures . . . SA2
Training and validating . . . SA3
Training and validation in practice . . . SA6
Large genomes . . . SA6
Small genomes . . . SA8
Prediction . . . SA9
Implementation . . . SA10
On the models' ability to generalise to unseen data . . . SA10
LSTM50S . . . SA11
LSTM50P . . . SA12
A cautious estimate . . . SA13
Yeast . . . SA17
Segmentation of output . . . SA18
Fourier transformations . . . SA18

"Simple" models **SA20**
k central models . . . SA21
Markov model . . . SA22
Likelihood ratio tests . . . SA23

Neural networks

Generalities

Neural networks take numeric input and apply arithmetic operations to it to produce a likewise numeric output. To make a neural network applicable to DNA strings, it is necessary to map the four bases (and any ”wild cards”) to the numeric world. For this we use ”one-hot encoding” of the four letters as follows: A is mapped to (1,0,0,0), C to (0,1,0,0), G to (0,0,1,0) and T to (0,0,0,1). Thus the four letters are replaced by four linearly independent vectors in four dimensional real space, $\mathbb{R}^4$; this independence is important for not introducing numeric correlation between the four letters. The encoding is extended to strings of letters by applying it to each letter in the string. Importantly, this means that our ’word-encoding’ is defined by a linear extension, so that e.g. the word ’AACG’ is closer to ’AACT’ than to ’AGCT’. Thus a string of length L is mapped into 4*L dimensional real space, $\mathbb{R}^{4L}$. The computations carried out in the first layer of a network applied to such strings therefore take place in this vector space.

The input to a model is a one-hot encoded genomic context (two flanks, merged or not). The output is a four dimensional real vector following the same encoding and for which the entries are positive and sum to 1. The output can then be interpreted as a probability distribution over the four letters; so for instance the first entry is the probability of having the letter A as output given the input. This property of the output is the result of letting the final layer of the network have a ”soft-max” function as its activation. Activation functions are elsewhere set to the ”rectified linear”, sometimes referred to as ReLu.

Architectures

We experimented with various types of architectures and settings, training all on human DNA (assemblies hg19 and hg38).

First we tried classic feed forward networks (best accuracy of about 46 $\%$) and then convolutional networks (best accuracy of about 48 $\%$). We also experimented with merging a network and a central model, resulting though in unstable training (data not included).

Upon moving to Long Short term Memory (LSTM) networks [1] accuracy could be improved to pass the 50$\%$ mark. In all these the LSTM layers are applied bi-directionally: moving forward (5’ to 3’) on the left-hand flank part and backward on the right-hand flank part. With the aim to include a kind of ”word en-

coding” we let the networks be initiated by one or two convolutional layers, with filters of size 3 or 4 and applied with a stride of 1. The number of filters was very simply inspired by the number of words in the four letter alphabet of length equal to the filter size. Following this word encoding, which is applied separately to the two flanks, the LSTM layers follow (mostly just two layers). The final LSTM layer outputs its final node value from the left-hand flank part and from the right-hand flank part. These are concatenated and fed into a final dense layer, intended for the purpose of obtaining a condensed representation of DNA context. The final layer outputs four positive real numbers summing to 1 as described above.

The convolutional-LSTM models contain LSTM[flank size] in their names, where flank size is counted in number of bases (as elsewhere in this work).

The architecture of the model that we refer to as LSTM50 is laid out graphically in Figure SA1. This model architecture is used for all the species we have considered (human, mouse, fruit fly, yeat and zebrafish).

The remaining models were only applied to the human genome; for all results are based on assembly hg38.

The architecture of the model referred to as LSTM200 is almost identical to that of LSTM50, except that LSTM200 has an extra word encoding convolutional layer and uses flanks of sizes 200 to either side, shown in Figure SA2. The model called LSTM200early is in architecture identical to LSTM200; LSTM200early is simply the model taken out after 15 rounds of the training for LSTM200 (similarly the yeast and drosophila models LSTM50early are obtained early in the training of their LSTM50’s). The model named LSTM50S has the same convolutional layers as LSTM200, but uses contexts of flank size 50 (as the LSTM50), so is an LSTM50-LSTM200 intermediate. The model named LSTM50P has architecture identical to that of LSTM50, but differ in the training and testing (more below).

All the convolutional-LSTM models have about 2.1 to 2.3 million trainable parameters.

Training and validating

To train one of our networks it is fed with pairs of (context, label) where label is the base at the (mid) position of the context. Formally, if we let x denote the genome sequence, at position i the context of flank size k is

$$c_i(k) = x_{i-k}, x_{i-k+1}, \ldots, x_{i-1}, x_{i+1}, x_{i+2}, \ldots, x_{i+k} \tag{1}$$

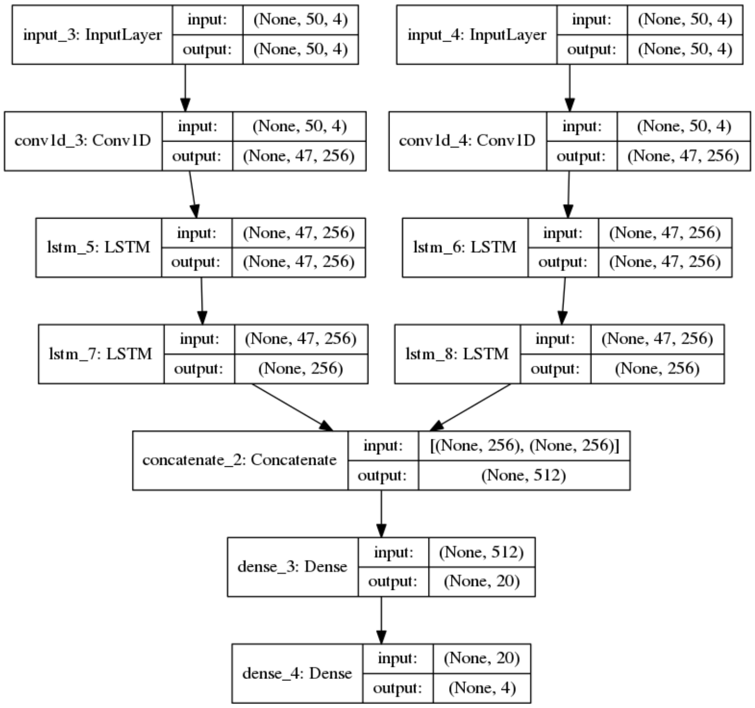

Figure SA1: Our convolutional-LSTM LSTM50. Flanks have size 50 and the LSTM layers are applied bi-directionally on the flanks, going left-to-right on the left-hand flank and vice verse on the right. The first LSTM layers each output a sequence of the same length as the input; the second LSTM layers each output the final position.

and the label is x_i. Please notice that the position of the context is *not* a part of the input. At some genomic positions the true base is unknown or uncertain, and a ”wild card”, typically the letter N, occurs there. If a context or its label contained a wild card it was disqualified from the training.

Training sets of such pairs, (context, label), were obtained by sampling from the entirety of the given genome (for convenience e.g. the first 3 billion positions of the human genome) or a part of it (see the description of LSTM50S and LSTM50P in Section On the models' ability to generalise to unseen data below). Throughout we used a single strand of the genome; we suppress this in what follows. The objective of the training was to fit the output probability distributions over the four bases (for all positions in the training set) as well as possible to the one-hot encoded targets (which can also be seen as probability distributions over

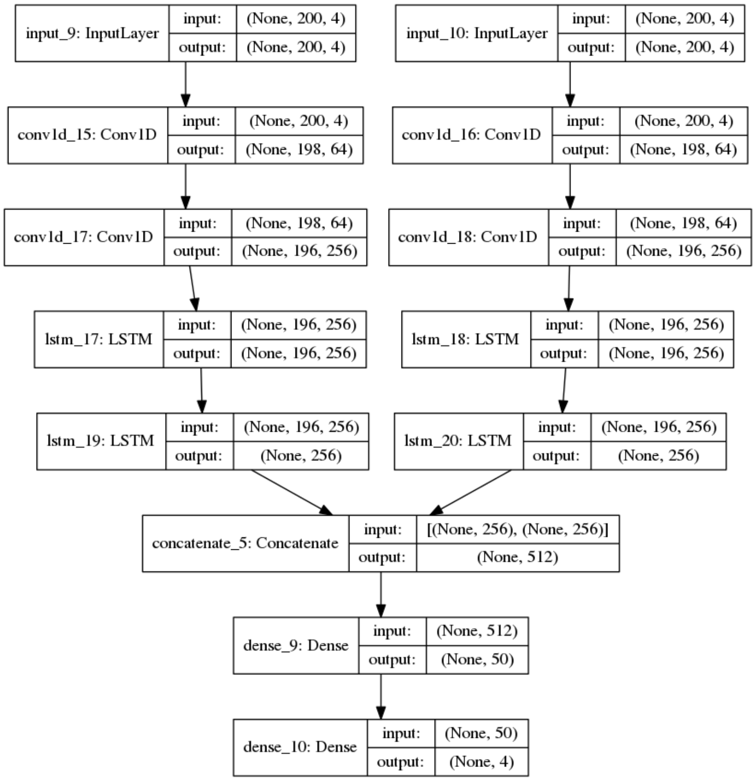

Figure SA2: Our convolutional-LSTM LSTM200. Similar to LSTM50 (above), but flanks have size 200 and the word encoding consists of two convolutional layers, the first encoding three letter words.

the four bases at the given position, viz. by placing the entire mass of 1 at the index of the true base). Formally the loss considered is the cross entropy of these distributions, called the categorical cross-entropy

$$loss = -\sum_{j} \sum_{m=1,2,3,4} t_{jm} \log(p_{jm}) \tag{2}$$

where the first sum is over the positions considered (indexed by j), m runs over the indexes of the one-hot encoding, t_{jm} is the target's one-hot encoding at index m (so e.g. if G is present at position j, t_{jm} is 1 for $m = 3$ and else zero) and p_{jm} is the model's probability for the letter corresponding to m (e.g. $m = 1$ it would be A). The loss is therefore equal to minus the log-likelihood of the model (the likelihood being given by the model's probability of the true base at any given position) and the objective is to minimize this loss.

Training and validation in practice

Except otherwise stated the training was run in ”rounds” of 5 million samples; at the end of each round the model was validated at that stage on a similarly sampled set of 1 million contexts. Each round consisted of 100 ”epochs”, one epoch being done in 100 steps of optimizing the model's parameters; each step in turn comprised a batch of 500 sampled contexts with corresponding labels. The full training consisted in completing up to 200 of such rounds (for the main models see Table SA1 below for the actual numbers; for the smaller genomes of yeast and fruit fly we used fewer rounds, see more later).

Large genomes

By the sizes of the genomes (human, mouse, zebrafish) of about 2.5-3 billion positions and the size of 200 training rounds of about 1 billion positions (each round being 5 million), the probability of a given position being included in the training would be roughly about one-third. As a result, a given training example is seen only slightly more than once on average; for concrete estimates see 'coverage' in Table SA1. Thus it seemed hardly necessary to keep validation and training sets disjoint (the former are used for the validation of the model after each training round; the real test of a model is had when applying it to new data, which here consists in about two-thirds of an entire genome). However, for all models except the LSTM200 (which was the first to be trained of the models presented here), the training and validation sets were separated by a 80-20 split: At initiation of the training 80 $\%$ of the total genomic positions considered were picked randomly as sampling material for the training sets, and the remaining 20 $\%$ were left for sampling the validation sets. In Figure SA3 we have monitored the performance of the main models throughout their training, showing validation results that steadily compare well to those of the training, revealing also that no overfitting was present. For more in depth considerations on the ability of the models to generalise to unseen data — also for the final test of applying the trained models to the full genomes – please see the Section On the models' ability to generalise to unseen data below.

The training of the LSTM200/LSTM200early was special in one further aspect. The trained model ought to have the following symmetry property: When applied to the reverse complement of a given context, its output must be the (reverse) complement of the original output. Formally, if $model(c) = b$ where c is a

model	#all	fraction	#rounds	#samples	#diff samples	coverage
LSTM200	2747445252	1.0	100	$1x10^9$	838221028	1.19
LSTM50	2747665597	0.8	177	$8.85x10^8$	728527509	1.21
LSTM50P	2179730830	1.0	200	$1x10^9$	802011938	1.25
LSTM50S	1383888064	0.8	185	$9.25x10^8$	627007586	1.48
LSTM200early	2747445252	1.0	16	$1.6x10^8$	155430262	1.03
mouseLSTM50	2389150131	0.8	194	$9.7x10^8$	760710935	1.28

Table SA1: Some sampling statistics for the training of the main models. #all: Number of all positions; fraction: The fraction of all positions included in the sampling for the training; #rounds: The number of training rounds; #samples per round: Number of samples drawn in each round; #samples: Total number of samples drawn in the training; #diff samples: expected number of different samples throughout the training; coverage: The expected number of times a given training sample was included. The expectations are computed using a binomial distribution with probability of being drawn equal to 1 divided by #all. The number of samples per round was 10 million for LSTM200/LSTM200early and 5 million for the other models.

context and b is the output base (label), then

$$model(\bar{c}) = \bar{b}$$

where $^-$ means reverse complement. To guide the training of a model towards having this property, one can let the training batches be invariant under reverse complement: By augmenting each batch of 500 training samples with the reverse complemented contexts and labels, the parameters were continuously updated on batches of 1000 samples, each batch invariant under taking reverse complement. For LSTM200/LSTM200early each training round then in fact counted 10 million samples, and the full training session ran therefore through 100 rounds and not 200.

For each full training session the model showing the best validation result during all rounds was picked out and used for further analysis. In all cases the best round was found in the late stages of the training (i.e. close to the final round). Of course, this does not apply to LSTM200early.

Small genomes

For the small genomes (yeast and fruit fly) we let the training continue far beyond only covering a relatively small fraction of the entire genomes, and then also considered models obtained early in this training as well as models at much later stages. For both species we split the input genome sequence in a training and a validation pool (80-20 $\%$) as described above. Thus for yeast we considered a model, called LSTM50early, obtained after the first repeat (5 million samples, slightly less than half the genome size) and a model, named LSTM50, obtained at 59 rounds/repeats (where the process will have covered the 80 $\%$ of the genome designated for training about 30 times). For the fruit fly we considered an early stage model, LSTM50early, at 12 repeats (the training material then covering about one third of the genome) and a late stage model, LSTM50, at 168 rounds/repeats (where the process will have covered the training material about 6 times). The results we report are for the early stage models unless otherwise stated. Results for both the early and the late stage models as well as plots monitoring the training process can be found in the Suppl. Tables and plots. The plots reveal that for yeast there is clear overfitting with a discrepancy between accuracy in the training and the validation of up to 2%. For fruit fly there is also some overfitting, but less severe (the discrepancy being less than 0.5%). The results for the late stage models could be interpreted as "best fits" to the data, but the overfitting is clearly undesired, since we want to capture general features in the data. (Despite the title, the aim of the paper is not really to predict the DNA. The aim is rather to fit models with the purpose of investigating – or revealing — structure in the DNA strings. The prediction task serves as a good frame in which to do this. We could have trained the models on e.g. the human genome far more extensively, while monitoring over-fitting. The slow growth in accuracy during training as is evident from the plots below, Fig. SA3 and time constraints kept us from pursuing further training on the large genomes).

On yeast we further trained models to investigate the training process. This is described in Section On the models' ability to generalise to unseen data below.

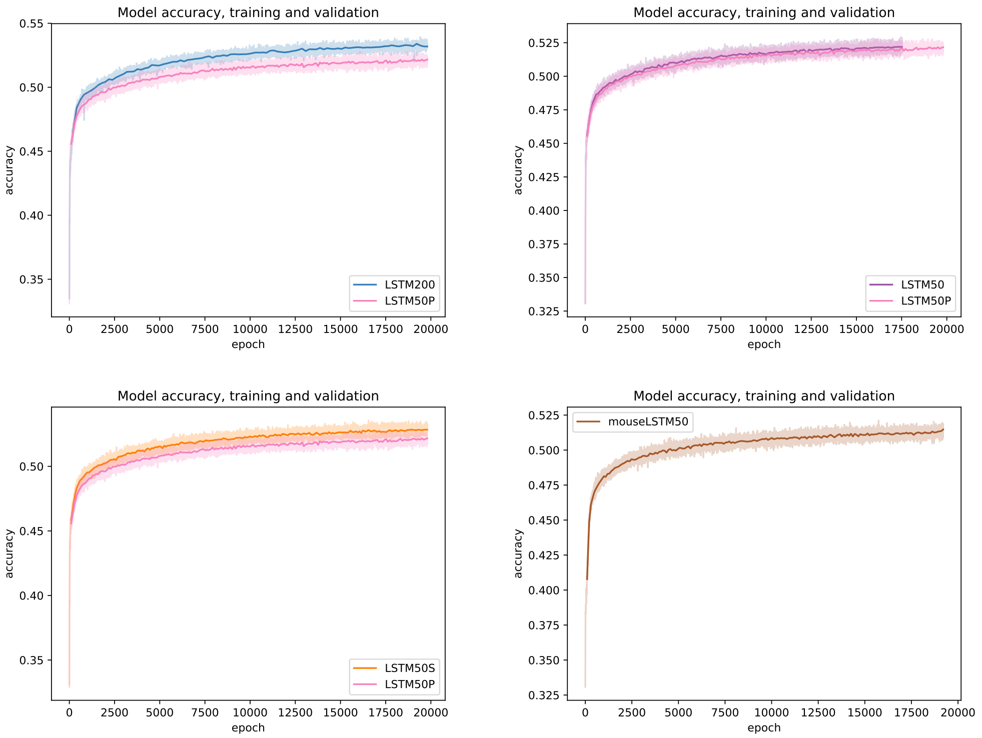

Figure SA3: Accuracy during training (dimmed) with validations (solid line) of the main convolutional-LSTMs. Except for the mouseLSTM50 case, the LSTM50P is shown in each plot for ease of comparison.

Prediction

After training, a model was applied to the complete single strand of the genome on which it was trained (unless otherwise stated): for every position the model was fed with the context and the four probabilities output by the model were kept (this was carried out in batches of 528 positions). The base predicted by the model was then set to the letter having the highest probability: at genomic position i the predicted base in one-hot encoding is

$$prediction_i = \mathbb{1}_{argmax_{j=1,2,3,4}(p_i(j))}$$

where $\mathbb{1}$ is short-hand for a ”boolean array on four entries” (so e.g. $\mathbb{1}_3$ is $(0, 0, 1, 0)$ i.e. the encoding of letter G).

For disqualified positions (i.e. if the context or label contained a non-ACGT letter — a wild card) the prediction was set to a (different) wild card. Disqualified positions were recorded and kept out of downstream computations, e.g. of accuracy and likelihood ratio tests. For a few of the human chromosomes a large heading section was disregarded, since it consists in N's (e.g. for chr22: the start position was set to 10500000; for more see the Supplementary on Data and data checks).

As an exception, LSTM50P was not applied to the full genome (see Section On the models' ability to generalise to unseen data below).

Implementation

We implemented the neural networks using Keras/Tensorflow [3], an open-source machine learning platform in Python. Among many great facilities, this includes automatic off-loading of core computations to Graphical Processing Units (GPUs), making the training process and predictions doable on a timescale of days, weeks or months. Our server ran with 12 GB RAM dedicated to the GPU, a Nvidia Tesla P100.

The training time varied considerably: for the low-complexity models, feed forward and convolutional, the training on the human genome took one to two days. The convolutional-LSTMSs took much more time to train, but highly influenced by the flanks size: for flanks of size 50 (LSTM50) the training took about 1 week; for flanks about 200 (LSTM200) some 3 weeks were spend. We observed that the last 2 weeks of training resulted in a gain in accuracy of about two percentages points. Applying the models to the full genome took about the same amount of time as the training.

On the models' ability to generalise to unseen data

As explained, we applied the models to the entire genomes, which includes all training data. The accuracy obtained may therefore be an exaggeration of the models' ability to predict on unseen data (true performance). The latter is often referred to as a model's ability to generalise. The exaggeration of the performance is due to test data, which is not truly unseen in the training: This can be training material included in the test material or, more generally, overlapping context in the training and test sets. Both are relevant in our case. Since we have framed our modelling as one of predicting and we certainly want to avoid overfitting, we

provide some reasoning and results to show that the obtained levels are notwithstanding fair estimates of the models' true performance, typically no more than 0.1 $\%$ point above a conservative accuracy estimate.

As mentioned, throughout the training the models' performance steadily generalised well to unseen data, as is clear from Figure SA3. However, only for the model LSTM50P was the validation data strictly disjoint from the training data; for the other models a validation context may intersect a training sample. The LSTM50P and also the LSTM50S that we now describe in more detail were trained and tested mainly for the purpose of addressing the overlapping-training-test data issue.

LSTM50S

LSTM50S was trained only on the odd-numbered chromosomes of hg38. The architecture of LSTM50S 'lies in between' that of LSTM50 and that of LSTM200 (see above). As expected, the performance of LSTM50S turned out to be only marginally better than that of LSTM50 (Fig. SA5 and Fig.SA6), and with no clear signs that the generalization to the even-numbered chromosomes was out of level with the performance on the odd-numbered chromosomes (results of LSTM50S are included with those of the other models in tables in the Suppl. Tables and plots). To make this clear, in Table SA2 and Figure SA4 we show the performance split on the odd/even-numbered chromosomes.

Evidently, all models are doing better on the odd-numbered chromosomes than on the even-numbered. The difference is though more pronounced for LSTM50S, which could be the result of a slight overestimation. On the other hand, LSTM50S was trained exclusively on the odd-numbered chromosomes and with a number of samples similar to that of the training of the other models. It is therefore expected that relative to the other models the performance of LSTM50S is slightly improved on the odd-numbered chromosomes, and slightly worsened on the even-numbered. Indeed, this appears to be the case, as is seen when comparing to LSTM50.

model/partition	odd	even	diff
LSTM200	0.5342	0.5281	0.0061
LSTM50	0.5237	0.5175	0.0062
LSTM50P	0.5224	0.5187	0.0037
LSTM50S	0.5251	0.5166	0.0085
LSTM200early	0.513	0.5066	0.0064

Table SA2: Accuracy aggregated on odd/even numbered chromosomes.

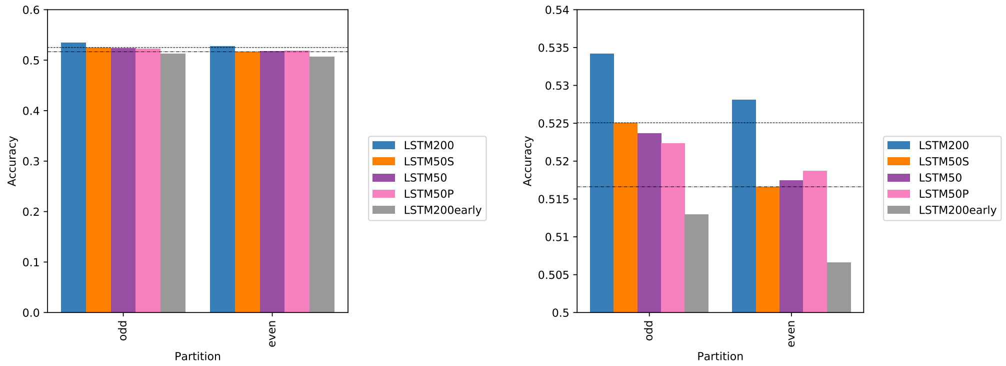

Figure SA4: Accuracy on odd/even numbered chromosomes of the models as indicated. To the left bar height shows the accuracy level; to the right the height indicates performance above 50%. The top (lower) horizontal dotted line indicates the accuracy level of LSTM50S on the odd(even)-numbered chromosome.

LSTM50P

Our comparison of LSTM50S to the other models is mildly confounded by the fact that the chromosomes have some structural differences (which the figures above also reflect), and due to the models' differences. We therefore trained a model, LSTM50P, having architecture identical to that of LSTM50 but trained differently: For its training we split the genome by dividing it in alternating sized segments of 400 kb and 100 kb; we then let the training data consist of the union of all 400 kb segments, while the test data was the union of all the 100 kb segments. (For both sets we concatenated these segments with a wildcard letter in between them; artificial contexts spanning a junction were therefore disqualified). This resulted in a 4:1 training:test data split, and with no contexts in the test set overlapping any

contexts in the training set. Also, the size of the training data set for LSTM50P equals what we used for LSTM50. For the validations of LSTM50P we used the test data set (as mentioned, the training is independent of the validation, which is only used for monitoring the development).

The training of LSTM50P followed very closely that of LSTM50 (Fig. SA3). One may notice that the validations of the two models show the same behaviour: The validation curves (solid) fall in the middle of the training track (dimmed).

The performance of LSTM50P on the total test set turned out virtually identical to its training level (the figures are shown in Table SA3). Thus, at these amounts of training, LSTM50P appears to generalise perfectly. Further, as expected, LSTM50P and LSTM50 perform very similarly across the chromosomes (Fig. SA5) and the resulting overall accuracy levels are very close (Table SA3). From these findings, we conclude that an amplification of the accuracy of LSTM50 stemming from shared or overlapping contexts between test and training is hardly there.

A cautious estimate

The results from LSTM50S and LSTM50P should suffice to substantiate our case. The accuracy level of LSTM50P can serve as an unbiased estimate of the performance of LSTM50 on the full genome, and the level of LSTM50S on the even-numbered chromosomes is also unbiased. However, we do not have a full set of unbiased estimates for all models. To make up for the gap, we computed a simple conservative estimate of the true performance. The accuracy level we have obtained on the full genome is a combination of two parts: the accuracy on all samples of the training and a level which is the performance on the remainder, which is the desired estimate. This gives also a fair estimate of the true performance in case of overlapping contexts in training and test sets, since we have just found that these hardly give rise to inflation. The accuracy level on the training part we set cautiously to the (average) performance throughout the last training round (i.e. the round at which the model's parameters are defined). This is clearly cautious since it assumes that the model will attain the applied 'training accuracy' level on all training samples (each training sample is visited only slightly more often than once). We then know two-of-three in this equation, which easily gives us the desired estimate

$$\text{accuracy} = w * \text{training accuracy} + (1 - w) * \text{estimate}, \tag{3}$$

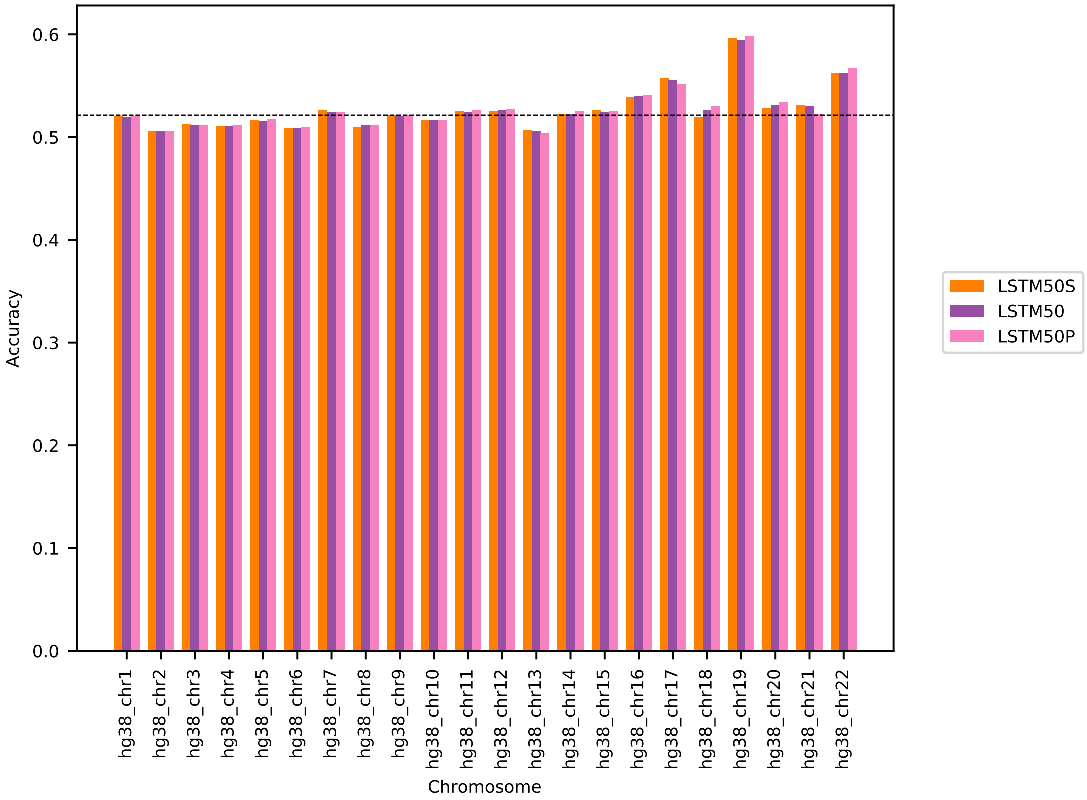

Figure SA5: Accuracy comparison of LSTM50S, LSTM50 and LSTM50P across the human autosomal chromosomes (hg38). For LSTM50S and LSTM50 the levels are obtained on the full chromosomes, while for LSTM50P the levels are obtained on the dedicated test data set. The dotted line indicates the average performance of LSTM50P (0.5214; average performance of LSTM50/S is 0.5206/0.5208). All values can be found in Table 1 in the Supplementary Tables and Plots.

where w is the fraction of different training samples to the number of positions in the genome at which a prediction was made. In Table SA3 and Fig. SA7 below we show the resulting estimates; the error bars reflect only the uncertainty of the 'training accuracy' and is given by the standard deviation on the accuracy through the 100 epochs of the last round of training (each epoch covers 50000 samples). As can be seen the accuracy (full genome) is typically about 0.1% point above

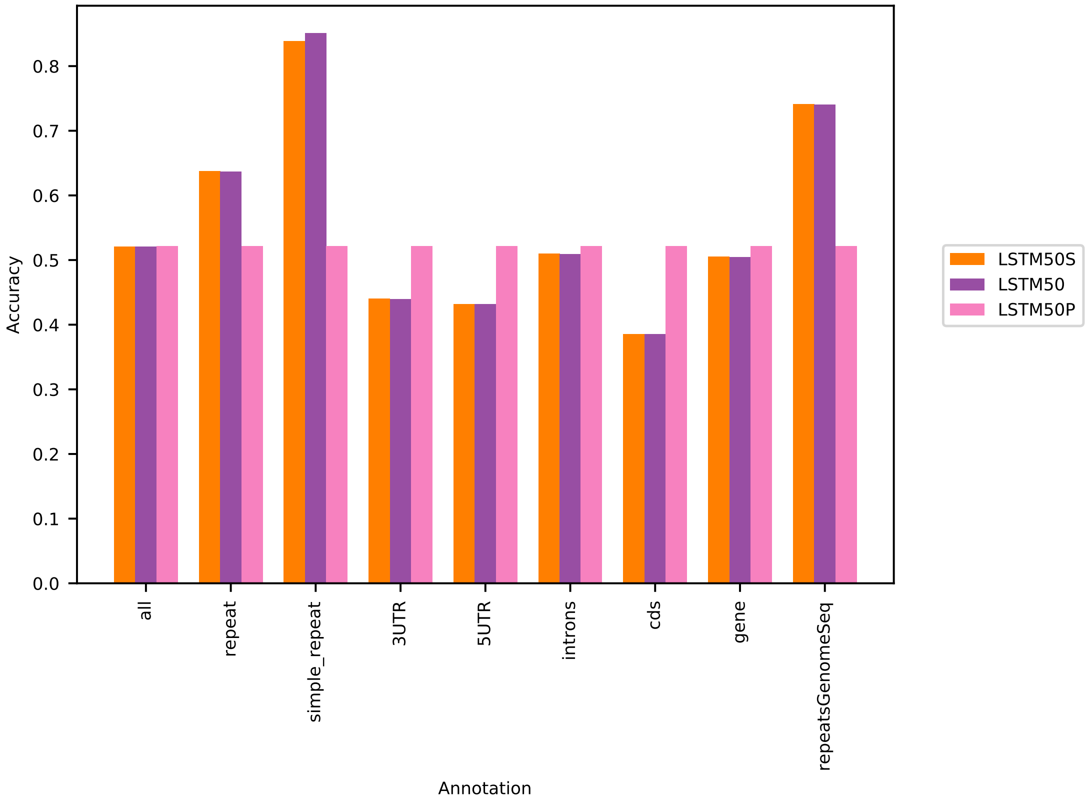

Figure SA6: Model accuracy comparison by annotation. For LSTM50P we only show the overall average on the dedicated test data set; for LSTM50S and LSTM50 the levels are obtained on the full genomes. All values can be found in Table 2 in the Supplementary Tables and Plots.

the estimated level. For LSTM50S the numbers are based on the odd-numbered chromosomes, and the difference is about 0.25% (on the full genome the estimate would then be about 0.13% lower than the obtained accuracy, since there is of course no difference on the even-numbered chromosomes). The bigger difference between odd/even numbered chromosomes for LSTM50S vs. the other models could stem from overestimation and reflect differences in how many times the training examples were visited: In the case of LSTM50S/odd-numbered chromosomes the training samples were visited slightly more often than for the other models (see 'coverage' in Table SA1); the estimate is then less cautions, and the

overestimation for LSTM50S/odd-numbered chromosomes could be higher than for the other models. However, the apparently perfect ability of LSTM50P to generalise points to that the difference is more likely due to the fact that LSTM50S has learned the characteristics of the odd-numbered chromosomes better than the other models and at the same time those of the even-numbered worse.

**model/level**	**acc last train**	**acc last val**	**acc all**	**acc estimate**
LSTM200	0.533	0.5319	0.5312	0.5304
LSTM50	0.5219	0.5219	0.5206	0.5201
LSTM50P	0.5212	0.5217	0.5214	-
LSTM50S	0.5281	0.5281	0.5251	0.5226
LSTM200early	0.5104	0.512	0.5098	0.5098
mouseLSTM50	0.5128	0.5148	0.5081	0.5059

Table SA3: The accuracy obtained in training ('acc last train', the average accuracy over the defining training round), validation ('acc last val', the validation done after the defining training round), on the full genome ('acc all'). Last our cautious estimate ('acc est'). The values for LSTM50S are based only on the odd-numbered chromosomes; the 'acc all' for LSTM50P is obtained on its dedicated test data set.

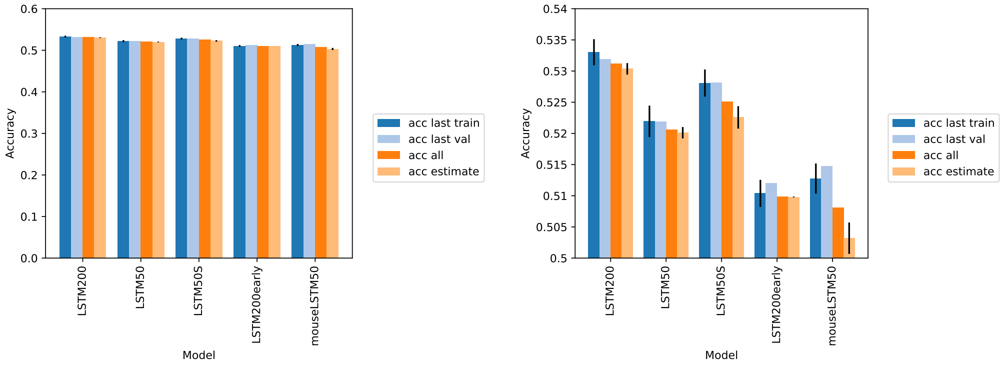

Figure SA7: The accuracy (y-axis) obtained in training ('acc last train', the average accuracy over the defining training round), validation ('acc last val', the validation done after the defining training round), on the full genome ('acc all') and our cautious estimate ('acc est'). To the left, bar height indicates the accuracy level, while to the right the height shows the level above 0.5. For LSTM50S only the values on the odd-numbered chromosomes were used.

Yeast

Since on yeast we noticed that the training appeared unstable, we also looked into that training process by training some dedicated models. We here show three training processes with models identical in architecture to LSTM50: LSTM50P on a truly split genome (just like LSTM50P on human DNA that we have just described); LSTM50Q and LSTM50R with training-validation split just as LSTM50. We let the training rounds consist of only 10 epochs (instead of the 100 used with LSTM50). A plot of the processes is shown in SA8. The training may result in quite severe overfitting (LSTM50P/R) or very vague overfitting (LSTM50Q) and the resulting accuracies (validation) vary by several percentage points. A fair estimate of the prediction-accuracy is about 40% (LSTM50P). More importantly, overfitting can be monitored even though validation data may overlap training data (LSTM50R). This was also seen with LSTM50 on yeast and LSTM50 on fruit fly (plots in Suppl. Tables and plots) and further supports that only little information may be passed via overlapping contexts.

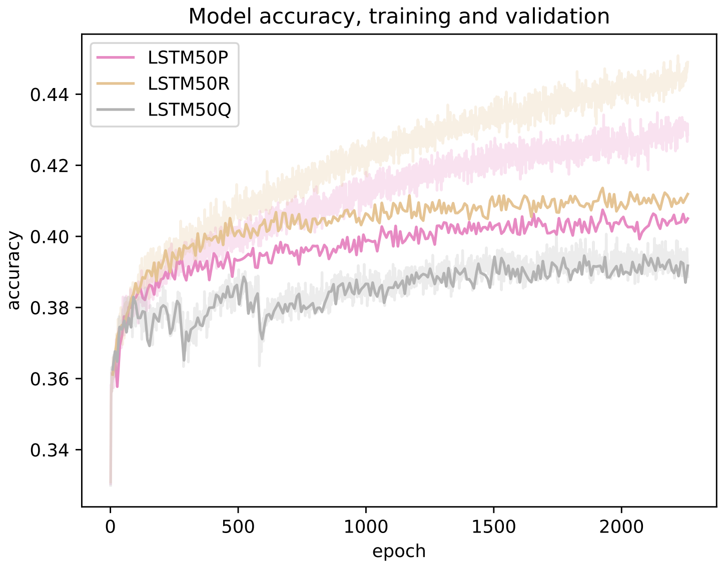

Figure SA8: Accuracy during training (dimmed) with validations (solid line) of LSTM50's on yeast. LSTM4P is trained on a truly split genome so that no validation context overlaps a training context. For LSTM50Q/R the training and validation contexts may overlap (but not perfectly).

Segmentation of output

A model's combined output (array of probabilities or predictions) consists of an array of length equal to the covered genome string. To handle this, we partitioned the input genome string in adjacent, disjoint 1 Mb segments, as many as could fit into the genome string. We handled the chromosomes separately. Thus all output (probabilities/predictions) and information related to it (e.g. a boolean array recording if a position was qualified or not) were segmented in the very same way.

Fourier transformations

Fourier transformation was done on every 1 Mb segment for which more than 90 % of the positions were qualified. The transformation was carried out on the model's output and on arrays encoding GC/AT content. In the first case, the input to the transformation was the model's probability of the reference base at every

position (the reference base being the base in the genome at a given position). For the GC/AT content case, the input consisted in arrays having a 1 at every G and C, and zero elsewhere (also in 1 Mb segments). These arrays had then no bearing on the models, but followed the same segmentation.

Since the input arrays have only real-value entries, the Fourier transforms are Hermitian: the coefficient of a given frequency is the complex conjugate of the coefficient for the corresponding negative frequency. For this reason we only show (the norms of) the coefficients in the positive frequency range.

In general the Fourier coefficients turned out not to reveal any structure that met the eye (an example is shown in Figure SA9, left). However, when summing up the 2-norm of the coefficients within a sliding window (in ”frequency space” that is), peaked patterns occurred in several cases (an example is shown in Figure SA9, mid). The aggregation amounts to taking the 2-norm of the part of the transform given by the frequencies within each window (apart from a factor of the square root of two: for a given window we ought to include also the corresponding window of negative frequencies, but by Pythagoras' theorem and the Hermitian property of the transform the 2-norm of these two parts equals the square root of two times the 2-norm of the coefficients in one of the two windows). A window size of 1000 and a step size of 100 were generally used (larger genomes), while shorter for yeast (window size 100 and step size 10).

The plots of the Fouriers transformation results are truncated for the very first low frequencies and from high frequencies; thus we show plots covering various frequency ranges, e.g. [200, 45000] and [40000, 120000]. The reason is that the left out parts appear uninteresting and if including them the signals would ”drown” in the scale.

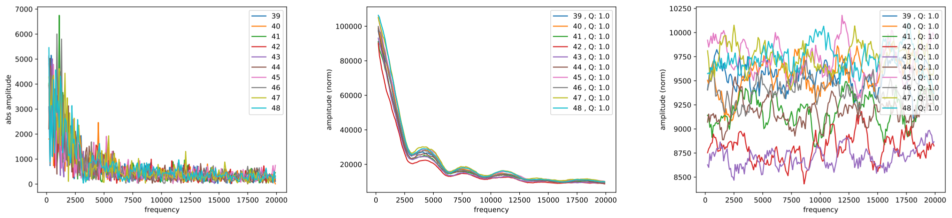

Figure SA9: Plots of outputs from Fouriers transforming the reference-base probability for segments 40 to 49 (1 Mb each) of chromosome hg38_chr19 according to LSTM200. From the left: absolute values of the Fourier coeffcients; L2-norm of Fourier coeffecients in a sliding window of 1000 (freqeuncies); absolute value of the Fourier coeffecients when shuffling the input. The segment numbers along with the fraction of qualified positions are indicated in the legend.

To check that the obtained patterns were not numeric artifacts, we randomized the input arrays in several ways. First, input arrays were shuffled; upon Fourier transformation no patterns were seen (Figure SA9, right). Second, we randomized various parts of the arrays: all disqualified positions; 50 $\%$ randomly picked and 100 $\%$ of all positions (Figure SA10). At a given position the randomization was done by replacing the value with the value of a randomly picked position in the same segment. Randomizing at all disqualified positions had only very little effect (this of course only hits the segments having disqualified positions). Randomizing all positions (a type of shuffling) wiped out the signal, while the signal survived randomizing even as much as half of all positions, albeit weakened. We made similar observations for Fourier output on the GC/AT content. This clearly points to that the periodicity signals are not computational artefacts. The differences seen in the results found for the genomes of various organisms also underpin this. Further, dramatic differences in patterns were seen in some few segments of chromosomes of the human genome (hg38); these segments appeared to overlap with centromeric regions. Similar behaviour was not seen in the case of the mouse genome, fitting well with the fact that in mouse the centromeres are placed at the ends of the chromosomes (the short arms being essentially absent).

”Simple” models

We tested our neural networks (the LSTMs) against the ”simple models” of [2]. For ease of implementation we ran the k central models for $k = 3, 4, 5$ to serve

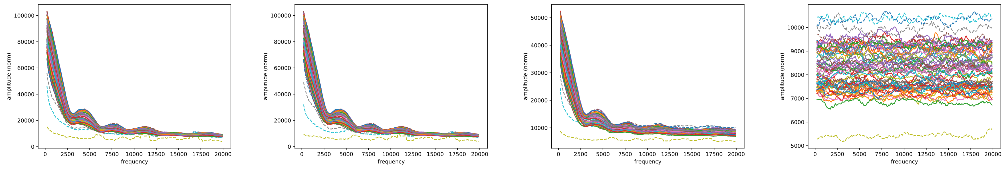

Figure SA10: Plots of outputs from Fouriers transforming the reference-base probability of chromosome hg38_chr20 according to LSTM200, as well as randomized as described in th text. Furthest left, the untouched L2-norm of Fourier coeffecients in a sliding window of 1000 (frequencies). Then follows the randomized versions: randomized at all disqualified positions; next at 50 % randomly picked positions and finally at all positions (a type of shuffling). The segment numbers along with the fraction of qualified positions are indicated in the legend.

this paper (the $k = 5$ central model interpolated from the $k = 4$ central model); the results could easily be stored in Python dictionaries. The run of the Markov model $k = 14$ was though taken from [2], the results being read into Python from a flat file.

We here include for completeness details of how the simple models were estimated; for more please consult [2]. Importantly, for each of these models the parameters were estimated using the entire genome.

k central models

Recalling the notation introduced above, $c_i(k)$ stands for the context of size k, symmetric around the position i (chromosome, coordinate) in the reference genome, and with x_i denoting the central base. To estimate the conditional probability of base b at position i, we use the counts $n(b|c_i)$ of the occurrences of b in the same context (omitting the k for ease of reading) throughout the reference genome (on both strands):

$$P(b|c_i) = \frac{n(b|c_i) - \delta_{b,x_i}}{N(c_i) - 1}, \tag{4}$$

where

$$N(c_i) = \sum_b n(b|c_i).$$

and where we use the Kronecker δ_{b,x_i}, which is 1 if $x_i = b$ and otherwise 0, to ensure that we only count *other* contexts, when estimating probabilities at position i.

This can be seen as a leave-one-out procedure. In particular, the estimate depends on the reference base at the considered position as well as the context. To obtain an estimate that is independent of the reference base at the position, a natural way to proceed is to consider the average of the four base-dependent estimates over all occurrences of the given context. It is easy to see that this average turns out to be equal to the estimate that includes all positions

$$\bar{P}(b|c) = \frac{n(b|c)}{N(c)}.$$

This is the estimation we have used for the k central models here.

For large contexts, the counts become small and thus the probabilities cannot be reliably estimated. To interpolate between different orders of the model, we use regularization by pseudo-counts obtained from the $k-1$ model. Specifically, for order k, we define pseudo-counts

$$r(b|c_i(k)) = \alpha P(b|c_i(k-1)),$$

where α is the strength of pseudo-counts. Now the model of order k is estimated as before, but using the actual counts plus pseudo-counts,

$$P(b|c_i(k)) = \frac{n(b|c_i(k)) + r(b|c_i(k))}{N(c_i(k)) - 1 + \alpha}.$$

We use this with $\alpha = 100$ for estimating the $k = 5$ central model and for the $k = 14$ Markov model that we now turn to.

Markov model

In a Markov model of order k, the probability of a base is conditioned on the k previous bases. If we redefine the k-context in (1) to be the k previous bases,

$$c_i(k) = x_{i-k}, x_{i-k+1}, \ldots, x_{i-1},$$

we can use exactly the same formulation as above. The context size is not $2k$ letters as above, but only k letters. Therefore, one can estimate Markov models up to sizes around $k = 14$ for the human genome, and we used a model interpolated from $k = 8$ to $k = 14$ analogously to the central interpolated model described above.

Estimating in this way a ”forward” Markov model from both strands of the chromosome gives for a given position two probability distributions over the four bases: one for the forward strand and one for the reverse strand. Our final Markov probabilities are the average between these.

Likelihood ratio tests

Likelihood ratio (LR) tests for non-nested models [4] were carried out for the LSTMs vs. the simpler models. We also implemented a nested-models version. The tests were implemented in Python; we checked our implementation by comparing the non-nested model test to a nested-model test, when both were applied to the nested-models case (two k central models).

For the case of nested models, what is calculated is (minus one times) the logarithm of the ratio of two terms, one representing the 'test' model's aptitude to fit the data ('numerator') and one representing the base model's aptitude ('denominator'). Each term is the product of the model's likelihoods of the true base across all sampled positions. By taking the logarithm the ratio becomes a difference and the products results in sums, so we compute the test size

$$\textit{LR} = -\sum_n \log(p_{\textit{test model}}(x_n)) + \sum_n \log(p_{\textit{base model}}(x_n)) \tag{5}$$

where the sums extend over all sampled positions, and x_n is the actual base at position n. For nested models LR is chi-squared distributed. The intuition is that the better the test model is at predicting the true bases than the base model (higher likelihoods), the bigger is the test size.

For the case of non-nested models, under the null hypothesis of equivalent models, the average test size LR/N, with N the number of samples, is normally distributed with mean zero and a variance that can be estimated by

$$var_{LR} = v/n - (LR/n)^2 \tag{6}$$

where v is obtained similarly to LR from the models' likelihoods

$$v = \sum_n (\log(p_{\textit{test model}}(x_n)) - \log(p_{\textit{base model}}(x_n))^2 \tag{7}$$

The final test size $LR/sqrt(N * var)$ is then normally distributed with mean zero and variance 1 (i.e. a "z-score").

The likelihood ratio tests of LSTM200 (as test model) vs. each of the simpler models (as base model) were carried out on each chromosome of the human genome GRCh38; each of these tests ran on a random sample of 10 $\%$ of the chromosome's positions. Results were recorded so that a test figure for the entirety of the chromosomes could be computed too. This allowed also to see if the test figures' behaviour with respect to sample size was as expected [4] (upon a rejection

of the null-hypothesis of equally well performing models, the test size should go to $+\infty$ for sample sizes going to $+\infty$ in the case that the ”numerator” model was superior). For a plot of the test sizes per chromosome see the Supplementary Plots and Tables.

References

[1] Hochreiter S. and Schmidhuber J. (1997). Long Short-term Memory. *Neural computation*, **9**, 1735–80.

[2] Liang, Y., Grønbæk, C., Fariselli, P., and Krogh, A. (2022). Context dependency of nucleotide probabilities and variants in human dna. *BMC Genomics*, **23**.

[3] Tensorflow (2018). `https://www.tensorflow.org`.

[4] Vuong, Q. (1989). Likelihood ratio tests for model selection and non-nested hypotheses. *Econometrica*, **57**, 307–33.
