# Supplementary material for: Context dependent prediction in DNA sequence using neural networks
Source: PeerJ. 2022 Sep 20;10:e13666. doi: 10.7717/peerj.13666 (PMC9504454; doi:10.7717/peerj.13666)
Supplement: Supplemental Information 2 [file peerj-10-13666-s002.pdf]

Supplementary B: Context dependent prediction in DNA sequence using neural networks. Data and data checks.

Christian Grønbæk, Yuhu Liang, Desmond Elliott, and Anders Krogh

April 17, 2022

Contents

Genome data **SB1**
Human SB1
Mouse SB5
Zebrafish SB6
Fruit fly SB8
Yeast SB8

Test of sampling in training procedure **SB8**

Genome data

Reference genomes for human, mouse, fruit fly, zebrafish and yeast were downloaded at public sites. Below we give details for each organism along with some statistics useful in conjunction with the predictions and accuracy computations for which we have reported results.

Human

We downloaded assembly GRCh38 from [2] in ”soft masked” format at the site https://www.ncbi.nlm.nih.gov/genome/?term=human (the same sequence can be

found as hg38 on [3]). Only the primary sequences were used. Sequences for the individual chromosomes (chr1-chr22) were obtained by splitting the full sequences. The (soft) repeat masking is done by the WindowMasker (the hg38 sequence at [3] probably has a slightly different repeat masking). We refer to this repeat matter as ”repeat masked” or ”RepeatGenomeSeq”.

The files (bed-format) we used for annotations (simple repeats, repeats, UTR’s, gene and coding section, cds) were downloaded from [3] at the interactive site https://genome.ucsc.edu/cgi-bin/hgTables.

To check the hg38 chromosome sequences we got in this way (and which we have used in all our analyses) we compared them base-by-base to the ones that can be had directly from the UCSC genome browser [3]. Concretely we downloaded the sequences of the individual chromosomes that we used for checking at http://hgdownload.soe.ucsc.edu/goldenPath/hg38/chromosomes/ (July, 2020).

In a second test aimed to check the soundness of our encoding, we report the number of different bases found upon inverting the one-hot encoded chromosome strings back to the genomic alphabet.

The results of these two tests can be seen in Table SB1, which reports the differences in number of bases. Discrepancies are seen but so few that they cannot harm our analyses.

Table SB2 shows the ”statistics” of the input to our predictions and accuracy computations. Qualified positions are those for which a context given by 50 bases (LSTM50 setting; for LSTM200 we used 200 bases) to both sides plus the position itself does not contain a non-ACGTacgt letter. The ”fraction of qualified” is the fraction of qualified positions relative to the whole genome sequences (for each chromosome). The number of segments is the number of segments (here 1 Mb long) for which predictions were made. For a few chromosomes (13, 14, 15 and 22) the number of segments does not match the length of the genome sequence; for these chromosomes there are long initial stretches of N’s and we chose to not do predictions on them (all these positions are disqualified so the prediction is irrelevant). Therefore, for these chromosomes, the predictions start at positions later than 0; the start positions were chr13: 16 million, chr14: 16 million, chr15: 17 million, chr22: 10.5 million. The low number of segments for these chromosomes reflects covering only these ”truncated” sequences. These four chromosomes also show the lowest fraction of qualified positions, which though overall is high (generally above 90 %).

The remaining numbers in Table SB2 give the fraction of the qualified positions having the named annotation. These were used for computing the accuracy of the prediction restricted to these annotated parts of the genome.

**chr**	**length**	**#diffs 1-hot**	**#diffs bases**
hg38_chr1	248956422	2	2
hg38_chr2	242193529	7	9
hg38_chr3	198295559	5	7
hg38_chr4	190214555	0	0
hg38_chr5	181538259	0	0
hg38_chr6	170805979	1	1
hg38_chr7	159345973	4	4
hg38_chr8	145138636	0	0
hg38_chr9	138394717	3	3
hg38_chr10	133797422	30	36
hg38_chr11	135086622	0	0
hg38_chr12	133275309	3	3
hg38_chr13	114364328	3	3
hg38_chr14	107043718	0	0
hg38_chr15	101991189	0	0
hg38_chr16	90338345	1	1
hg38_chr17	83257441	11	12
hg38_chr18	80373285	0	0
hg38_chr19	58617616	0	0
hg38_chr20	64444167	0	0
hg38_chr21	46709983	3	3
hg38_chr22	50818468	4	5

Table SB1: Human genome, assembly GRCh38/hg38. Length of the autosomal chromosomes (nr of bases) and number of different bases in the two checks (see text).

chr	#qualified	fraction qualified	#seg-ments	repeat masked	simple repeat	repeat	cds	introns	3UTR	5UTR	gene	all
hg38_chr1	229495936	0.922	248	0.37	0.005	0.518	0.014	0.602	0.023	0.009	0.635	1.0
hg38_chr2	240353008	0.992	242	0.356	0.001	0.493	0.01	0.593	0.016	0.007	0.616	1.0
hg38_chr3	197853613	0.998	198	0.371	0.033	0.514	0.009	0.623	0.016	0.006	0.645	1.0
hg38_chr4	189591912	0.997	190	0.382	0.029	0.518	0.007	0.546	0.013	0.005	0.564	1.0
hg38_chr5	180772919	0.996	181	0.377	0.002	0.515	0.008	0.56	0.015	0.006	0.581	1.0
hg38_chr6	169326743	0.991	170	0.37	0.028	0.502	0.008	0.381	0.018	0.01	0.355	1.0
hg38_chr7	158627095	0.995	159	0.387	0.051	0.514	0.008	0.404	0.018	0.01	0.373	1.0
hg38_chr8	144685300	0.997	145	0.364	0.007	0.514	0.006	0.419	0.017	0.01	0.377	1.0
hg38_chr9	121438540	0.877	138	0.372	0.044	0.518	0.009	0.378	0.019	0.011	0.349	1.0
hg38_chr10	132497298	0.99	133	0.364	0.003	0.5	0.008	0.388	0.018	0.01	0.358	1.0
hg38_chr11	134450920	0.995	135	0.37	0.052	0.527	0.014	0.583	0.021	0.01	0.614	1.0
hg38_chr12	132861272	0.997	133	0.388	0.003	0.53	0.012	0.604	0.024	0.008	0.635	1.0
hg38_chr13	97620716	0.854	98	0.368	0.04	0.493	0.005	0.312	0.015	0.008	0.277	1.0
hg38_chr14	90558669	0.846	91	0.38	0.045	0.517	0.009	0.444	0.023	0.013	0.41	1.0
hg38_chr15	83653532	0.82	84	0.373	0.053	0.515	0.011	0.464	0.028	0.016	0.433	1.0
hg38_chr16	81569398	0.903	90	0.382	0.065	0.518	0.013	0.426	0.031	0.02	0.408	1.0
hg38_chr17	82658588	0.993	83	0.403	0.008	0.521	0.022	0.625	0.031	0.013	0.67	1.0
hg38_chr18	79812216	0.993	80	0.383	0.087	0.51	0.006	0.508	0.016	0.006	0.527	1.0
hg38_chr19	57830142	0.987	58	0.467	0.109	0.599	0.034	0.607	0.043	0.017	0.669	1.0
hg38_chr20	63576097	0.987	64	0.367	0.017	0.537	0.012	0.527	0.019	0.01	0.557	1.0
hg38_chr21	39378764	0.843	41	0.391	0.022	0.518	0.007	0.515	0.016	0.01	0.537	1.0
hg38_chr22	38832574	0.764	40	0.394	0.107	0.536	0.017	0.574	0.034	0.016	0.62	1.0

Table SB2: Human, GRCh38/hg38. Statistics on input to the prediction. For explanation of the columns see the text.

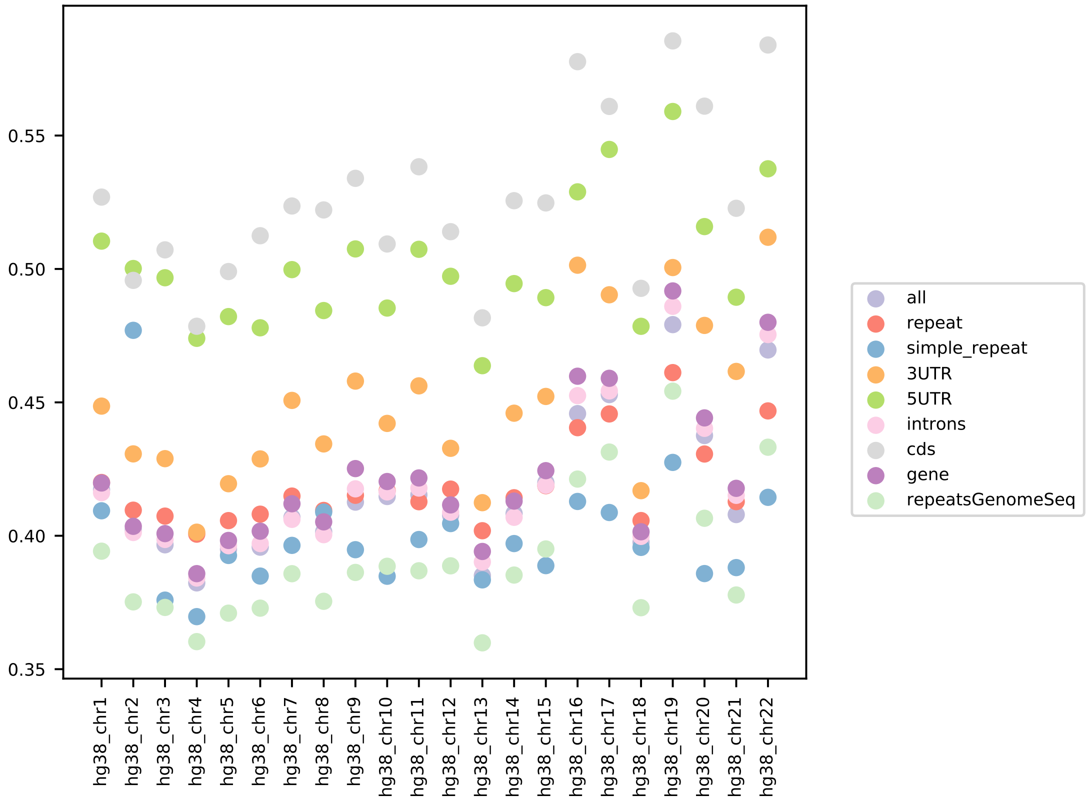

Figure SB1: GC content per chromosome and annotation type for the human reference genome, assembly GRCh38/hg38.

Finally, for the human genome, Figure SB1 and Table SB3 show the GC content per annotation type. The coding sections (cds) and 5UTRs have the highest GC content; in the whole genomic sequence the fraction is about 41 %.

Mouse

The soft-masked mouse genome assembly GRCm38 (mm10) was downloaded from [1] at site ftp://ftp.ensembl.org/pub/release-101/fasta/mus_musculus/dna/. As with the human genome the file was split into files (sequences) for individual chromosomes.

We carried out the two same tests for the mouse chromosomes that we did for

chr	all	repeat	simple repeat	repeat masked	cds	gene	introns	3UTR	5UTR
hg38_chr1	0.4173	0.42	0.4094	0.3942	0.5269	0.4198	0.4162	0.4486	0.5104
hg38_chr2	0.4023	0.4096	0.477	0.3752	0.4957	0.4035	0.4012	0.4307	0.5002
hg38_chr3	0.3966	0.4074	0.3759	0.3731	0.5072	0.4008	0.3986	0.4289	0.4967
hg38_chr4	0.3823	0.4006	0.3697	0.3603	0.4785	0.3858	0.3842	0.4013	0.474
hg38_chr5	0.3949	0.4057	0.3926	0.371	0.499	0.3982	0.3963	0.4195	0.4821
hg38_chr6	0.3957	0.4081	0.3849	0.3729	0.5125	0.4017	0.3971	0.4288	0.4779
hg38_chr7	0.4069	0.4149	0.3964	0.3858	0.5236	0.412	0.4061	0.4507	0.4998
hg38_chr8	0.4015	0.4095	0.4089	0.3754	0.5221	0.4052	0.4004	0.4344	0.4844
hg38_chr9	0.4126	0.4152	0.3948	0.3863	0.534	0.4252	0.4176	0.458	0.5075
hg38_chr10	0.4147	0.4169	0.3848	0.3886	0.5094	0.4203	0.4164	0.4421	0.4853
hg38_chr11	0.4154	0.4128	0.3986	0.3869	0.5383	0.4217	0.4179	0.4562	0.5074
hg38_chr12	0.4076	0.4175	0.4046	0.3887	0.514	0.4116	0.4088	0.4328	0.4973
hg38_chr13	0.3851	0.4019	0.3835	0.3599	0.4817	0.3941	0.3902	0.4124	0.4637
hg38_chr14	0.4083	0.4142	0.3971	0.3853	0.5256	0.413	0.4069	0.4459	0.4945
hg38_chr15	0.4199	0.4188	0.3888	0.3951	0.5248	0.4244	0.4189	0.4522	0.4893
hg38_chr16	0.4458	0.4405	0.4129	0.4212	0.5777	0.4598	0.4525	0.5014	0.5289
hg38_chr17	0.4529	0.4457	0.4087	0.4314	0.5609	0.459	0.4543	0.4903	0.5448
hg38_chr18	0.3975	0.4057	0.3956	0.373	0.4927	0.4015	0.3998	0.4169	0.4785
hg38_chr19	0.4791	0.4612	0.4275	0.4542	0.5855	0.4918	0.486	0.5005	0.559
hg38_chr20	0.4376	0.4306	0.3858	0.4066	0.561	0.4442	0.4403	0.4788	0.5159
hg38_chr21	0.4079	0.4128	0.3881	0.3778	0.5227	0.4178	0.4151	0.4616	0.4894
hg38_chr22	0.4696	0.4468	0.4144	0.4332	0.5839	0.48	0.4754	0.5119	0.5375
All	0.4093	0.4152	0.3961	0.3854	0.527	0.4153	0.4113	0.448	0.5012

Table SB3: Human, GRCh38/hg38. Fraction of GC content per annotation.

the human and no differences were seen. The chromosome sequences used for the comparison were downloaded from [3] site http://hgdownload.soe.ucsc.edu/goldenPath/mm10/chromosomes/ (July, 2020).

Table SB4 reports the "statistics" for the input to the predictions and accuracy computations, just as Table SB2 for the human case. As can be seen the fraction of qualified positions is high throughout.

Zebrafish

The reference assembly GRCz11 (soft masked for showing repeats) was downloaded from [2], at site https://ftp.ncbi.nlm.nih.gov/genomes/refseq/vertebrate_other-/Danio_rerio/all_assembly_versions/GCF_000002035.6_GRCz11/, the concrete file being GCF_0000020- 35.6_GRCz11_genomic.fna.gz. As for the other genomes,

chr	length	#qualified	fraction qualified	#seg-ments	repeat masked	all
m38_chr1	195471971	191532614	0.98	195	0.457	1.0
m38_chr2	182113224	178311077	0.979	182	0.426	1.0
m38_chr3	160039680	156397328	0.977	160	0.465	1.0
m38_chr4	156508116	151745996	0.97	156	0.463	1.0
m38_chr5	151834684	147181940	0.969	151	0.442	1.0
m38_chr6	149736546	145749271	0.973	149	0.447	1.0
m38_chr7	145441459	141510898	0.973	145	0.476	1.0
m38_chr8	129401213	125309169	0.968	129	0.427	1.0
m38_chr9	124595110	120762514	0.969	124	0.435	1.0
m38_chr10	130694993	126469919	0.968	130	0.445	1.0
m38_chr11	122082543	118745645	0.973	122	0.413	1.0
m38_chr12	120129022	116892948	0.973	120	0.44	1.0
m38_chr13	120421639	116798804	0.97	120	0.444	1.0
m38_chr14	124902244	120638761	0.966	124	0.441	1.0
m38_chr15	104043685	100652515	0.967	104	0.433	1.0
m38_chr16	98207768	94911106	0.966	98	0.436	1.0
m38_chr17	94987271	90816441	0.956	94	0.448	1.0
m38_chr18	90702639	86849245	0.958	90	0.435	1.0
m38_chr19	61431566	57873940	0.942	61	0.419	1.0

Table SB4: Mouse genome, assembly GRCm38/mm10. Statistics on input to the predictions.

the full (primary) sequence was subsequently split, resulting in 25 chromosomes used for predictions.

Table SB5 reports the "statistics" for the input to the predictions and accuracy computations, just as Table SB2 for the human case. As can be seen the fraction of qualified positions is high throughout (> 96 %).

Fruit fly

The reference genome assembly was downloaded from [2], at https://ftp.ncbi.nlm-.nih.gov/genomes/genbank/invertebrate/Drosophila_melanogaster/all_assembly_versions, file: GCA_000001215.4_Release_6_plus_ISO1_MT and subsequently split so as to have the individual chromosome sequences.

Table SB6 reports the "statistics" for the input to the predictions and accuracy computations, just as Table SB2 for the human case. As can be seen the fraction of qualified positions is high throughout (> 97 %) except for the shortest chromosome, chr4.

Yeast

The yeast genome assembly R64 was downloaded from [4] at site http://sgd-archive.yeastgenome.org/sequence/S288C_reference/genome_releases/. As with the human genome the file was split into files (sequences) for individual chromosomes.

We carried out the two same tests for the yeast chromosomes that we did for mouse and human; no differences were seen. The chromosome sequences used for the comparison were downloaded from [3] site https://hgdownload.soe.ucsc.edu/-goldenPath/sacCer3/chromosomes/ (July, 2020).

The simple repeats annotation sequence was downloaded from [3].

Table SB7 reports the "statistics" for the input to the predictions and accuracy computations, just as Table SB2 for the human case. As can be seen, with one exception (chr6) the fraction of qualified positions is high throughout.

Test of sampling in training procedure

This section is dedicated to a test of the uniformity of the sampling used in the training of the neural networks. Since the sampling was not used for the pre-

chr	length	#qualified	fraction qualified	#seg- ments	repeat masked	all
GRCz11_chr1	59578282	58886847	0.988	59	0.473	1.0
GRCz11_chr2	59640629	58842337	0.987	59	0.494	1.0
GRCz11_chr3	62628489	61876776	0.988	62	0.479	1.0
GRCz11_chr4	78093715	75126176	0.962	78	0.525	1.0
GRCz11_chr5	72500376	71826576	0.991	72	0.493	1.0
GRCz11_chr6	60270059	59851582	0.993	60	0.493	1.0
GRCz11_chr7	74282399	73827529	0.994	74	0.477	1.0
GRCz11_chr8	54304671	53908128	0.993	54	0.494	1.0
GRCz11_chr9	56459846	55874391	0.99	56	0.483	1.0
GRCz11_chr10	45420867	44904680	0.989	45	0.491	1.0
GRCz11_chr11	45484837	44869208	0.986	45	0.488	1.0
GRCz11_chr12	49182954	48818150	0.993	49	0.49	1.0
GRCz11_chr13	52186027	51879672	0.994	52	0.488	1.0
GRCz11_chr14	52660232	51895223	0.985	52	0.486	1.0
GRCz11_chr15	48040578	47860510	0.996	48	0.492	1.0
GRCz11_chr16	55266484	54883113	0.993	55	0.497	1.0
GRCz11_chr17	53461100	52857727	0.989	53	0.491	1.0
GRCz11_chr18	51023478	50812317	0.996	51	0.488	1.0
GRCz11_chr19	48449771	47888635	0.988	48	0.485	1.0
GRCz11_chr20	55201332	54860117	0.994	55	0.494	1.0
GRCz11_chr21	45934066	44874023	0.977	45	0.494	1.0
GRCz11_chr22	39133080	38867105	0.993	39	0.475	1.0
GRCz11_chr23	46223584	45868239	0.992	46	0.488	1.0
GRCz11_chr24	42172926	41871251	0.993	42	0.485	1.0
GRCz11_chr25	37502051	36875129	0.983	37	0.494	1.0

Table SB5: Zebrafish, GRCz11. Statistics on input to the predictions.

chr	#qualified	fraction qualified	#seg-ments	all
r6.18_chrX	22935030	0.974	23	1.0
r6.18_chr2L	22999550	0.978	23	1.0
r6.18_chr2R	24992450	0.988	25	1.0
r6.18_chr3L	27880890	0.992	28	1.0
r6.18_chr3R	31972678	0.997	32	1.0
r6.18_chr4	999950	0.742	1	1.0

Table SB6: Fruit fly, r6.18. Statistics on input to the predictions.

chr	length	#qualified	fraction qualified	#seg-ments	simple repeat	all
R64_chr1	230218	199950	0.869	2	0.017	1.0
R64_chr2	813184	799950	0.984	8	0.005	1.0
R64_chr3	316620	299950	0.947	3	0.008	1.0
R64_chr4	1531933	1499950	0.979	15	na	1.0
R64_chr5	576874	499950	0.867	5	na	1.0
R64_chr6	270161	199950	0.74	2	na	1.0
R64_chr7	1090940	999950	0.917	10	na	1.0
R64_chr8	562643	499950	0.889	5	na	1.0
R64_chr9	439888	399950	0.909	4	na	1.0
R64_chr10	745751	699950	0.939	7	na	1.0
R64_chr11	666816	599950	0.9	6	na	1.0
R64_chr12	1078177	999950	0.927	10	na	1.0
R64_chr13	924431	899950	0.974	9	na	1.0
R64_chr14	784333	699950	0.892	7	na	1.0
R64_chr15	1091291	999950	0.916	10	na	1.0
R64_chr16	948066	899950	0.949	9	na	1.0

Table SB7: Yeast, R64. Statistics on input to the predictions.

dictions, this is to some extent only a ”nice-to-know”: were the sampling non-uniform over the genome only the training of the models could be harmed.

The test was done as follows. As explained in Supplementary Methods the training of the models were done in series of ”repeats” (”rounds” or ”big epochs”); each repeat consisted of 100 epochs, each in turn consisting in 100 steps of training batches of size 500. Thus each repeat uses 5 million samples. At the end of each repeat a validation was run based on 1 million samples. The training and validation samples were drawn from 4:1 division of the genomic sequence.

To test that this sampling was uniform, a full training sessions of 200 repeats was carried out, recording at every 10 repeats how many times each position was sampled (both for the training and for the validation). To reveal the uniformity the genome sequence was partitioned into adjacent windows of a set length. Two figures were then computed for every 10 repeats, based on all samples accumulated up to that repeat number:

1. Average occupancy: the average number of samples in the windows over the genome sequence

2. the standard deviation in the same set of occupancy numbers

This was carried out for two window sizes: 100000 and 1 million bases. Figure SB2 below shows these averages as a function of the repeat number (one average every 10 repeats) with bars at each measurement indication 10 times the standard deviation (for the sake of visibility). Clearly, the sampling appears to be uniform.

References

[1] Ensemble (2020). http://www.ensembl.org/.

[2] NCBI (2018). https://www.ncbi.nlm.nih.gov.

[3] UCSC (2018). https://hgdownload.cse.ucsc.edu.

[4] YeastGenome (2011). https://www.yeastgenome.org.

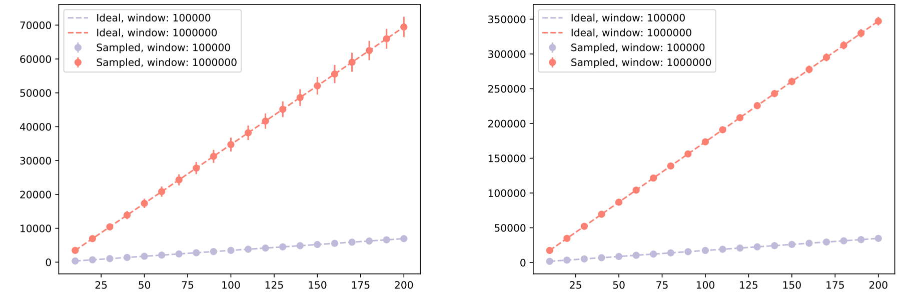

Figure SB2: Results of sampling test. The plot to the left shows the results for the validation samples, the one to the right the training samples. Dots represent the average number of samples (given window size) and the bars show 10 standard deviations in the set of sampling occupancy numbers per window (on which the averages are had).
