# Supplementary material for: Context dependent prediction in DNA sequence using neural networks
Source: PeerJ. 2022 Sep 20;10:e13666. doi: 10.7717/peerj.13666 (PMC9504454; doi:10.7717/peerj.13666)
Supplement: Supplemental Information 3 [file peerj-10-13666-s003.pdf]

Supplementary I: Context dependent prediction in DNA sequence using neural networks. Tables and additional plots.

Christian Grønbæk, Yuhu Liang, Desmond Elliott, and Anders Krogh

April 16, 2022

Contents

Human genome **SI2**

Other genomes **SI12**

Yeast, S.cerevisae SI12

Fruit fly, D.melanogaster SI13

Zebrafish, D.rerio SI15

Mouse SI16

Base composition **SI16**

Human genome

chr/model	LSTM200	LSTM50S	LSTM50	LSTM50P	LSTM200 early	mouse LSTM50
hg38_chr1	0.5299	0.5204	0.519	0.5205	0.5087	0.4347
hg38_chr2	0.5163	0.5055	0.5056	0.5062	0.4954	0.4278
hg38_chr3	0.5224	0.5128	0.5114	0.5118	0.4997	0.4258
hg38_chr4	0.5218	0.5107	0.5106	0.512	0.4987	0.4245
hg38_chr5	0.5267	0.5168	0.5155	0.5173	0.5042	0.4251
hg38_chr6	0.5193	0.5088	0.509	0.5098	0.4978	0.4272
hg38_chr7	0.5347	0.5257	0.5246	0.5243	0.5145	0.4355
hg38_chr8	0.5222	0.5097	0.5112	0.5112	0.4998	0.4264
hg38_chr9	0.5316	0.5218	0.5205	0.5216	0.5094	0.4327
hg38_chr10	0.5272	0.5161	0.5168	0.5167	0.5066	0.4331
hg38_chr11	0.5353	0.5252	0.5238	0.5259	0.5127	0.4283
hg38_chr12	0.5366	0.5248	0.5258	0.5275	0.5146	0.4341
hg38_chr13	0.5161	0.5064	0.5056	0.5037	0.4945	0.4237
hg38_chr14	0.533	0.5226	0.522	0.5253	0.5107	0.4315
hg38_chr15	0.5338	0.5262	0.5241	0.525	0.5137	0.4351
hg38_chr16	0.5496	0.539	0.5397	0.5404	0.5305	0.4484
hg38_chr17	0.5641	0.5572	0.5556	0.5517	0.5457	0.4557
hg38_chr18	0.5356	0.519	0.5258	0.5302	0.5148	0.4217
hg38_chr19	0.6018	0.5962	0.594	0.5982	0.5868	0.4811
hg38_chr20	0.5414	0.5286	0.5313	0.5336	0.5208	0.4357
hg38_chr21	0.5399	0.5307	0.5297	0.522	0.5181	0.4276
hg38_chr22	0.572	0.562	0.5619	0.5672	0.5519	0.4553

Table SI1: Accuracy of the predictions of the five models indicated per chromosome in human reference genome GRCh38 (hg38). For LSTM50P the values are obtained on the parts of the chromosomes dedicated for testing the model (see Suppl Methods).

anno/model	LSTM200	LSTM50S	LSTM50	LSTM50P	LSTM200 early	mouse LSTM50
all	0.5312	0.5209	0.5206	0.5212	0.5098	0.4321
repeat	0.6542	0.6375	0.637	na	0.615	0.4692
simple repeat	0.8546	0.8389	0.8508	na	0.8272	0.4968
3UTR	0.4461	0.4401	0.4396	na	0.4373	0.412
5UTR	0.4383	0.4319	0.4316	na	0.4291	0.4066
introns	0.5195	0.51	0.5091	na	0.4998	0.4356
cds	0.3896	0.3856	0.3858	na	0.3873	0.3847
gene	0.5146	0.5052	0.5044	na	0.4956	0.434
repeatsGenomeSeq	0.7504	0.7414	0.7409	na	0.7204	0.5389

Table SI2: Accuracy of the predictions of the five models indicated per annotation in human reference genome GRCh38 (hg38). For LSTM50P the values are obtained on the parts of the chromosomes dedicated for testing the model (see Suppl Methods).

chr	all	repeats	simple repeats	repeats masked	cds	gene	introns	3UTR	5UTR
hg38_chr1	0.5299	0.6501	0.795	0.7497	0.3897	0.5167	0.5218	0.4448	0.4424
hg38_chr2	0.5163	0.6375	0.764	0.7335	0.3793	0.5069	0.5102	0.4431	0.4325
hg38_chr3	0.5224	0.6412	0.8349	0.7379	0.3828	0.5094	0.5125	0.4387	0.438
hg38_chr4	0.5218	0.6361	0.8132	0.7276	0.3727	0.513	0.5161	0.4422	0.4314
hg38_chr5	0.5267	0.6484	0.9212	0.7426	0.3793	0.5115	0.5149	0.4328	0.4301
hg38_chr6	0.5193	0.6407	0.7976	0.7334	0.3849	0.5042	0.5116	0.4426	0.4257
hg38_chr7	0.5347	0.6607	0.8424	0.7522	0.3905	0.512	0.5184	0.438	0.4302
hg38_chr8	0.5222	0.6396	0.9364	0.7397	0.3885	0.5056	0.5114	0.4401	0.4328
hg38_chr9	0.5316	0.6536	0.8551	0.7517	0.3932	0.5123	0.5197	0.4385	0.4355
hg38_chr10	0.5272	0.6541	0.9247	0.7497	0.3822	0.5101	0.5175	0.4401	0.4253
hg38_chr11	0.5353	0.656	0.871	0.7598	0.3941	0.5135	0.5184	0.4428	0.4418
hg38_chr12	0.5366	0.6587	0.8815	0.7547	0.3849	0.5185	0.5231	0.4526	0.4401
hg38_chr13	0.5161	0.6364	0.8393	0.7257	0.378	0.4984	0.5041	0.4308	0.4308
hg38_chr14	0.533	0.6572	0.8555	0.7521	0.3902	0.5157	0.5233	0.4438	0.4355
hg38_chr15	0.5338	0.6608	0.8613	0.7606	0.3938	0.5174	0.5252	0.4483	0.4386
hg38_chr16	0.5496	0.6802	0.8498	0.7795	0.4069	0.533	0.5413	0.4595	0.4537
hg38_chr17	0.5641	0.7063	0.9502	0.7956	0.3997	0.5367	0.5443	0.4541	0.4516
hg38_chr18	0.5356	0.6686	0.9122	0.7631	0.3746	0.4988	0.5016	0.4398	0.4421
hg38_chr19	0.6018	0.7224	0.8437	0.8103	0.4092	0.5717	0.5844	0.4863	0.46
hg38_chr20	0.5414	0.6607	0.9244	0.7752	0.398	0.5129	0.5175	0.4483	0.4511
hg38_chr21	0.5399	0.6665	0.9667	0.7568	0.3851	0.503	0.5065	0.4416	0.437
hg38_chr22	0.572	0.7035	0.8626	0.8036	0.4057	0.5367	0.5438	0.4726	0.4624
All	0.5312	0.6542	0.8546	0.7504	0.3896	0.5146	0.5195	0.4461	0.4383

Table SI3: Accuracy of the predictions of LSTM200 per chromosome and annotation in human reference genome GRCh38 (hg38).

chromo	all	repeats	simple repeats	repeats masked	cds	gene	introns	3UTR	5UTR
hg38_chr1	0.519	0.6325	0.7811	0.7399	0.3857	0.5063	0.5111	0.4385	0.4359
hg38_chr2	0.5056	0.6193	0.7548	0.7231	0.3761	0.4967	0.4999	0.4368	0.4254
hg38_chr3	0.5114	0.623	0.8337	0.7276	0.3788	0.4987	0.5016	0.4327	0.4306
hg38_chr4	0.5106	0.6176	0.8116	0.7169	0.3694	0.5022	0.5051	0.4357	0.4248
hg38_chr5	0.5155	0.6299	0.9179	0.7319	0.3763	0.5007	0.5039	0.4265	0.4222
hg38_chr6	0.509	0.6234	0.8007	0.7236	0.382	0.4946	0.5016	0.4358	0.4192
hg38_chr7	0.5246	0.6442	0.8353	0.7428	0.3859	0.5026	0.5086	0.4323	0.4246
hg38_chr8	0.5112	0.6215	0.9432	0.7299	0.3858	0.4952	0.5007	0.4332	0.4256
hg38_chr9	0.5205	0.6355	0.8474	0.7415	0.3896	0.5021	0.5091	0.4323	0.4286
hg38_chr10	0.5168	0.6369	0.9299	0.7407	0.3789	0.5003	0.5074	0.4337	0.4194
hg38_chr11	0.5238	0.6376	0.8646	0.7498	0.3893	0.5024	0.5071	0.4356	0.4342
hg38_chr12	0.5258	0.6415	0.8727	0.7452	0.3817	0.5083	0.5126	0.4454	0.4326
hg38_chr13	0.5056	0.6185	0.8353	0.7154	0.3742	0.4885	0.4941	0.4243	0.4235
hg38_chr14	0.522	0.6394	0.8455	0.7418	0.3864	0.5054	0.5126	0.4371	0.4294
hg38_chr15	0.5241	0.6452	0.8676	0.753	0.3903	0.5075	0.5148	0.4418	0.4326
hg38_chr16	0.5397	0.6649	0.8441	0.7723	0.403	0.524	0.5318	0.4532	0.4475
hg38_chr17	0.5556	0.6938	0.9525	0.7899	0.3951	0.5281	0.5354	0.4479	0.445
hg38_chr18	0.5258	0.6527	0.9112	0.7547	0.3718	0.4888	0.4914	0.4334	0.434
hg38_chr19	0.594	0.7125	0.8387	0.805	0.4041	0.5637	0.5762	0.4789	0.4541
hg38_chr20	0.5313	0.6452	0.9294	0.7678	0.3932	0.5028	0.5072	0.4415	0.4438
hg38_chr21	0.5297	0.6502	0.9698	0.7474	0.3814	0.4929	0.4962	0.4357	0.4294
hg38_chr22	0.5619	0.6882	0.8503	0.7956	0.402	0.5273	0.5341	0.4664	0.4552
All	0.5206	0.637	0.8508	0.7409	0.3858	0.5044	0.5091	0.4396	0.4316

Table SI4: Accuracy of the predictions of LSTM50 per chromosome and annotation in human reference genome GRCh38.

chromo	all	repeats	simple repeats	repeats masked	cds	gene	introns	3UTR	5UTR
hg38_chr1	0.5087	0.6114	0.7752	0.7207	0.3877	0.4973	0.5016	0.4365	0.433
hg38_chr2	0.4954	0.5974	0.7464	0.7027	0.3771	0.4878	0.4906	0.4343	0.4227
hg38_chr3	0.4997	0.5993	0.8041	0.7049	0.3806	0.4889	0.4915	0.4301	0.4271
hg38_chr4	0.4987	0.5937	0.7879	0.6944	0.3701	0.4917	0.4944	0.4319	0.4206
hg38_chr5	0.5042	0.607	0.902	0.7107	0.3771	0.4908	0.4937	0.4249	0.4184
hg38_chr6	0.4978	0.6	0.7692	0.7016	0.3837	0.4859	0.4924	0.4328	0.4167
hg38_chr7	0.5145	0.6236	0.8196	0.7239	0.3887	0.4946	0.4998	0.4311	0.4227
hg38_chr8	0.4998	0.5986	0.8736	0.7076	0.3865	0.4862	0.4912	0.4303	0.4233
hg38_chr9	0.5094	0.6129	0.8271	0.7205	0.3906	0.4937	0.4999	0.4298	0.4271
hg38_chr10	0.5066	0.6154	0.8885	0.7208	0.3802	0.4923	0.4987	0.4312	0.4178
hg38_chr11	0.5127	0.6152	0.8493	0.7289	0.3911	0.4929	0.4971	0.4334	0.4314
hg38_chr12	0.5146	0.6194	0.8436	0.7247	0.3835	0.4991	0.503	0.4421	0.4305
hg38_chr13	0.4945	0.5951	0.8085	0.6938	0.376	0.4797	0.4848	0.4211	0.4195
hg38_chr14	0.5107	0.6163	0.8173	0.7198	0.388	0.4962	0.5027	0.4347	0.4273
hg38_chr15	0.5137	0.6239	0.8352	0.7326	0.3907	0.4991	0.5058	0.4383	0.429
hg38_chr16	0.5305	0.6462	0.828	0.7563	0.4044	0.517	0.524	0.4518	0.4458
hg38_chr17	0.5457	0.673	0.8985	0.7705	0.397	0.5217	0.5286	0.4472	0.4437
hg38_chr18	0.5148	0.6302	0.8845	0.7331	0.3724	0.4803	0.4827	0.4312	0.4304
hg38_chr19	0.5868	0.6987	0.8281	0.7927	0.4061	0.5583	0.5702	0.4766	0.4522
hg38_chr20	0.5208	0.6246	0.8945	0.7477	0.395	0.4951	0.499	0.4402	0.441
hg38_chr21	0.5181	0.6266	0.9228	0.7245	0.382	0.4845	0.4875	0.4333	0.4264
hg38_chr22	0.5519	0.6681	0.823	0.7765	0.4026	0.5204	0.5268	0.4641	0.453
All	0.5098	0.615	0.8272	0.7204	0.3873	0.4956	0.4998	0.4373	0.4291

Table SI5: Accuracy of the predictions of LSTM200early per chromosome and annotation in human reference genome GRCh38.

chromo	all	repeats	simple repeats	repeats masked	cds	gene	introns	3UTR	5UTR
hg38_chr1	0.4347	0.4742	0.5314	0.5474	0.3849	0.4348	0.4366	0.4122	0.4098
hg38_chr2	0.4278	0.4653	0.4544	0.5388	0.3749	0.4286	0.4298	0.4088	0.402
hg38_chr3	0.4258	0.4598	0.5285	0.531	0.3785	0.4262	0.4271	0.4073	0.4035
hg38_chr4	0.4245	0.454	0.5513	0.5241	0.3692	0.4257	0.4267	0.4065	0.3971
hg38_chr5	0.4251	0.4575	0.3977	0.5259	0.375	0.4263	0.4274	0.4052	0.3971
hg38_chr6	0.4272	0.4634	0.5607	0.5345	0.3794	0.4274	0.43	0.407	0.3974
hg38_chr7	0.4355	0.4755	0.5101	0.5429	0.3847	0.4352	0.4368	0.4098	0.404
hg38_chr8	0.4264	0.4597	0.3849	0.5322	0.3835	0.4274	0.4289	0.406	0.4019
hg38_chr9	0.4327	0.4696	0.5226	0.5405	0.3885	0.4348	0.4367	0.4085	0.4066
hg38_chr10	0.4331	0.4743	0.3858	0.5453	0.3776	0.4341	0.4369	0.4081	0.3997
hg38_chr11	0.4283	0.4602	0.4809	0.5292	0.3883	0.4299	0.4315	0.4093	0.4082
hg38_chr12	0.4341	0.472	0.4058	0.5422	0.3802	0.4359	0.4376	0.4113	0.4056
hg38_chr13	0.4237	0.4555	0.4924	0.5253	0.3728	0.425	0.4271	0.4006	0.3979
hg38_chr14	0.4315	0.468	0.4977	0.5361	0.3844	0.4328	0.4355	0.4085	0.4058
hg38_chr15	0.4351	0.4764	0.4908	0.5467	0.3888	0.4378	0.4403	0.4118	0.4055
hg38_chr16	0.4484	0.4977	0.5183	0.5683	0.3997	0.4543	0.457	0.4247	0.4203
hg38_chr17	0.4557	0.5075	0.3953	0.5702	0.3942	0.4583	0.4617	0.4206	0.4203
hg38_chr18	0.4217	0.4514	0.4263	0.5117	0.372	0.4255	0.4265	0.4072	0.4037
hg38_chr19	0.4811	0.5286	0.5106	0.5875	0.4053	0.4839	0.4903	0.439	0.4259
hg38_chr20	0.4357	0.4717	0.3914	0.5437	0.3918	0.439	0.4407	0.4163	0.4125
hg38_chr21	0.4276	0.4564	0.3638	0.5163	0.38	0.4306	0.432	0.4101	0.4043
hg38_chr22	0.4553	0.4968	0.469	0.5587	0.3998	0.4591	0.4622	0.4327	0.4237
All	0.4321	0.4692	0.4968	0.5389	0.3847	0.434	0.4356	0.412	0.4066

Table SI6: Accuracy of the predictions of mouseLSTM50 per chromosome and annotation in human reference genome GRCh38.

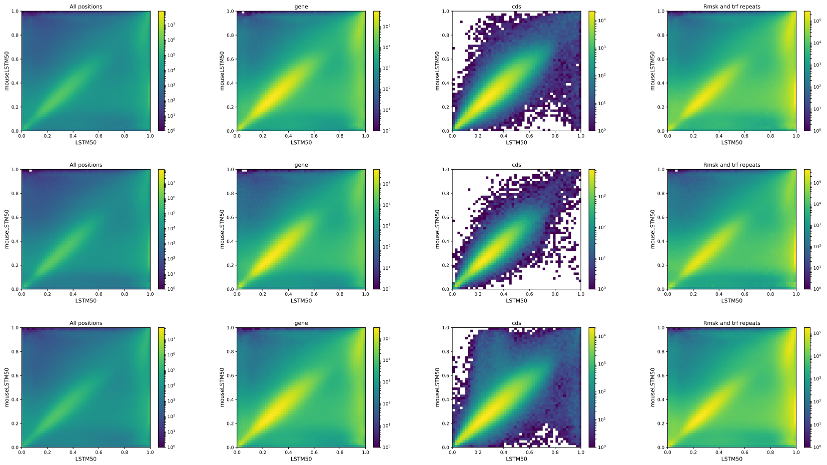

Figure SI1: LSTM50 vs mouseLSTM50. Density-plot of probabilities of reference bases in annotated parts of chromosomes of the human reference genome GRCh38 according to LSTM50 (x-axis) and mouseLSTM50 (y-axis). From the top: chromosome 17, 18, 19. Annotations from left to right: all positions, gene, cds, repeats.

**Model1**	**Model2**	**Test value**	**p-value**
LSTM200	k=3 central	1877.6	$< 10^{-20}$
LSTM200	k=4 central	1646.4	$< 10^{-20}$
LSTM200	k=5 central	1339.9	$< 10^{-20}$
LSTM200	Markov k=14	768.6	$< 10^{-20}$

Table SI7: Results of likelihood ratio tests. Model2 was used as base (”denominator”) in likelihood ratio test.

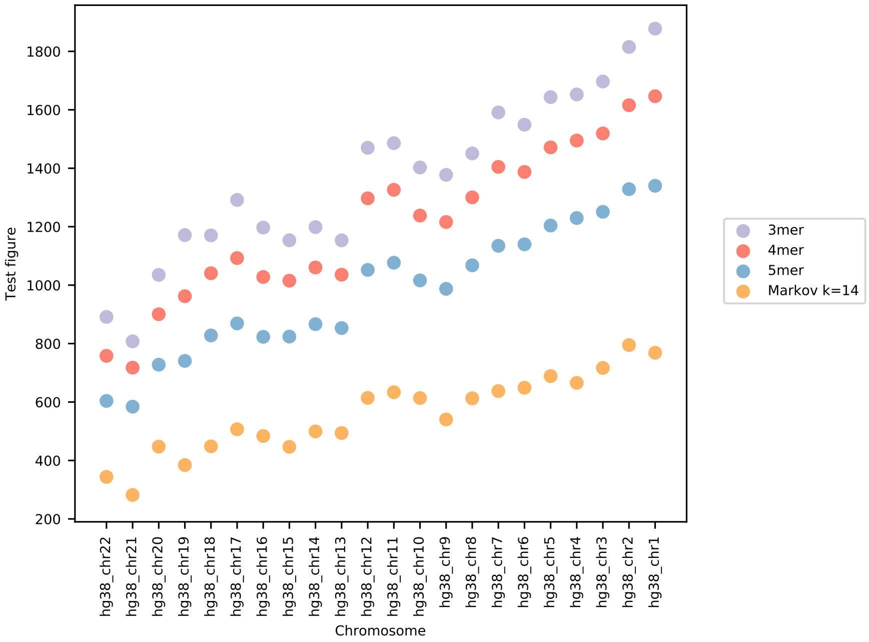

Figure SI2: Likelihood ratio test figures per chromosome for LSTM200 vs simpler models as indicated. Test values increase with the size of the sample size (equivalently the chromosome size) supporting the rejection of the null hypotheses of equally performing models [**?**].

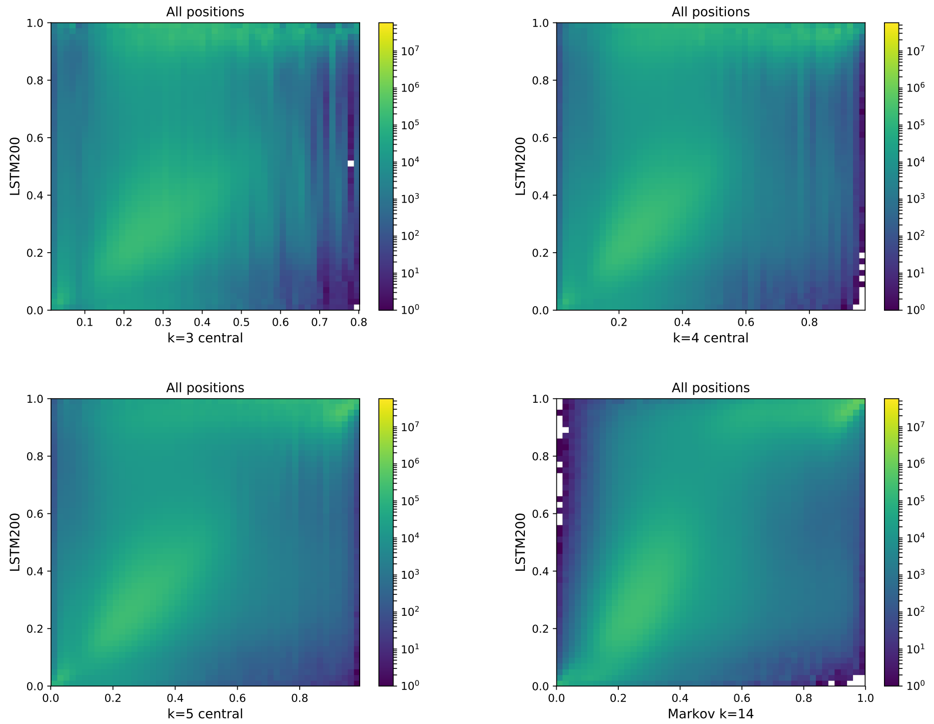

Figure SI3: LSTM200 (x-axis) vs simpler models (y-axis) on human hg38, chromosome 19. From left to right: central model for k=3,4,5 and Markov model, k=14. Each plot shows the density of the reference-base probabilities on chromosome 19 according to the named models. Please note that colors are not shared between the plots.

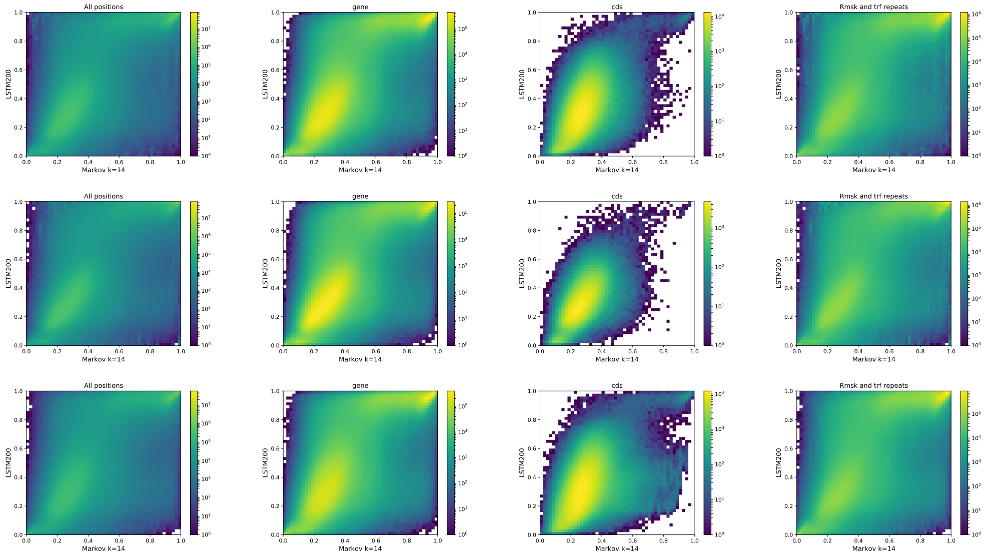

Figure SI4: Markov k=14 vs. LSTM200. Density-plot of probabilities of reference bases in annotated parts of chromosomes of the human reference genome GRCh38 according to the Markov k=14 model(x-axis) and LSTM200 (y-axis). From the top: chromosome 17, 18, 19. Annotations from left to right: all positions, gene, cds, repeats.

Other genomes

Yeast, S.cerevisae

chr/annotation	all	simple repeats
R64_chr1	0.369	0.4239
R64_chr2	0.372	0.4599
R64_chr3	0.3719	0.4591
R64_chr4	0.3744	na
R64_chr5	0.3681	na
R64_chr6	0.3705	na
R64_chr7	0.3728	na
R64_chr8	0.3704	na
R64_chr9	0.3707	na
R64_chr10	0.3705	na
R64_chr11	0.3731	na
R64_chr12	0.3704	na
R64_chr13	0.3733	na
R64_chr14	0.3693	na
R64_chr15	0.372	na
R64_chr16	0.3721	na
All	0.3718	0.4476

chr/annotation	all	simple repeats
R64_chr1	0.4221	0.6612
R64_chr2	0.416	0.6167
R64_chr3	0.4152	0.6394
R64_chr4	0.4209	na
R64_chr5	0.4169	na
R64_chr6	0.4194	na
R64_chr7	0.4191	na
R64_chr8	0.4077	na
R64_chr9	0.4101	na
R64_chr10	0.4152	na
R64_chr11	0.4081	na
R64_chr12	0.4201	na
R64_chr13	0.4192	na
R64_chr14	0.4133	na
R64_chr15	0.4135	na
R64_chr16	0.4192	na
All	0.4166	0.6373

Table SI8: Accuracy of the predictions of LSTM50early (left) and LSTM50 (right) on yeast genome R64. LSTM50early is identical to LSTM50, but less trained (see Suppl. methods).

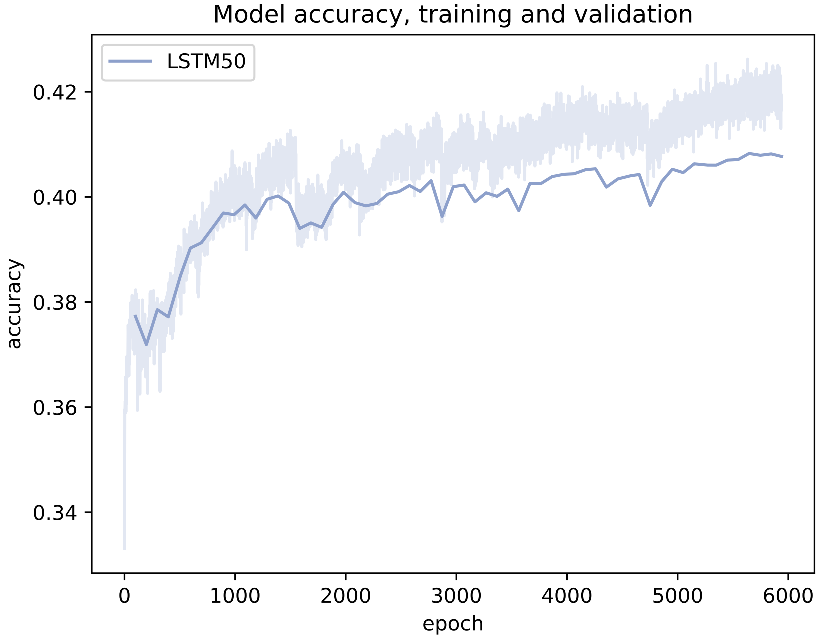

Figure SI5: LSTM50 on yeast. Accuracy during training (dimmed) with validations (solid line). Clear overfitting is seen.

Fruit fly, D.melanogaster

chr/annotation	all
r6.18_chrX	0.4359
r6.18_chr2L	0.4146
r6.18_chr2R	0.4105
r6.18_chr3L	0.4165
r6.18_chr3R	0.4183
r6.18_chr4	0.4157
All	0.4188

chr/annotation	all
r6.18_chrX	0.466
r6.18_chr2L	0.4425
r6.18_chr2R	0.4602
r6.18_chr3L	0.4604
r6.18_chr3R	0.4552
r6.18_chr4	0.4443
All	0.4568

Table SI9: Accuracy of the predictions of LSTM50early (left) and LSTM50 (right) on fruit fly genome dm6 (r6.18). LSTM50early is identical to LSTM50, but less trained (see Suppl. methods)

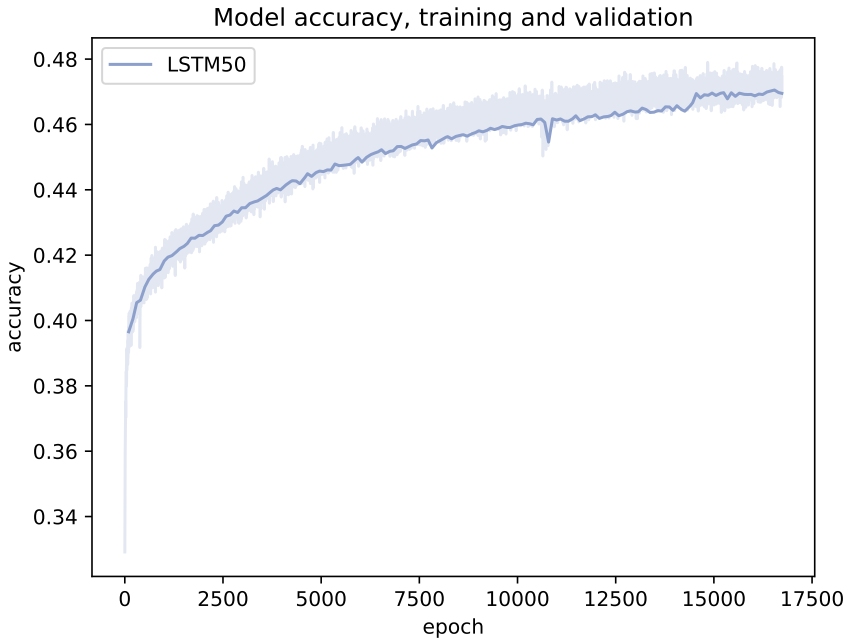

Figure SI6: LSTM50 on fruit fly. Accuracy during training (dimmed) with validations (solid line). Mild overfitting is seen.

Zebrafish, D.rerio

chr/annotation	all	repeats masked
GRCz11_chr1	0.5377	0.7019
GRCz11_chr2	0.5463	0.7067
GRCz11_chr3	0.5387	0.7004
GRCz11_chr4	0.5212	0.6504
GRCz11_chr5	0.5448	0.7043
GRCz11_chr6	0.5448	0.704
GRCz11_chr7	0.54	0.7066
GRCz11_chr8	0.5454	0.7048
GRCz11_chr9	0.5419	0.7053
GRCz11_chr10	0.5471	0.7088
GRCz11_chr11	0.5443	0.7054
GRCz11_chr12	0.5454	0.7066
GRCz11_chr13	0.5464	0.7098
GRCz11_chr14	0.5427	0.7062
GRCz11_chr15	0.5452	0.7052
GRCz11_chr16	0.5483	0.7081
GRCz11_chr17	0.5471	0.7098
GRCz11_chr18	0.5448	0.7075
GRCz11_chr19	0.5426	0.704
GRCz11_chr20	0.5458	0.7052
GRCz11_chr21	0.5459	0.7051
GRCz11_chr22	0.5366	0.6993
GRCz11_chr23	0.5455	0.7079
GRCz11_chr24	0.545	0.7085
GRCz11_chr25	0.5478	0.7089
All	0.5428	0.7024

Table SI10: Accuracy of the predictions of LSTM50 on zebrafish genome GRCz11.

Mouse

chr/annotation	all	repeats masked
m38_chr1	0.5136	0.6684
m38_chr2	0.5034	0.6643
m38_chr3	0.5188	0.6751
m38_chr4	0.5157	0.6664
m38_chr5	0.5105	0.6688
m38_chr6	0.51	0.6662
m38_chr7	0.5179	0.6653
m38_chr8	0.5036	0.6653
m38_chr9	0.5024	0.6573
m38_chr10	0.5085	0.6661
m38_chr11	0.4984	0.6588
m38_chr12	0.5059	0.6618
m38_chr13	0.5034	0.6573
m38_chr14	0.5077	0.6664
m38_chr15	0.5042	0.6618
m38_chr16	0.5043	0.6607
m38_chr17	0.5089	0.6617
m38_chr18	0.5026	0.6603
m38_chr19	0.4951	0.6526
All	0.5081	0.6644

Table SI11: Accuracy of the predictions of mouseLSTM50 on mouse reference genome GRCm38 (mm10).

Base composition

Species	A	C	G	T	Random guess acc
human (H.sapiens)	0.2952	0.2039	0.2048	0.2961	0.2583
yeast (S.cerevisae)	0.3098	0.1909	0.1906	0.3087	0.2640
fruit fly (D.melanogaster)	0.2900	0.2101	0.2099	0.2899	0.2564
mouse (M.musculus)	0.2915	0.2083	0.2083	0.291	0.2569
zebrafish (D.rerio)	0.3171	0.1830	0.1830	0.3169	0.2680

Table SI12: Frequency of the occurrence of each of the four bases for the species we have considered. The last row shows the accuracy level of predicting the base at a given position when applying a random guess adhering to the frequencies.
