# Supplementary material for: Context dependent prediction in DNA sequence using neural networks
Source: PeerJ. 2022 Sep 20;10:e13666. doi: 10.7717/peerj.13666 (PMC9504454; doi:10.7717/peerj.13666)
Supplement: Supplemental Information 10 [file peerj-10-13666-s010.pdf]

Supplementary IIe: Context dependent prediction in DNA sequence using neural networks. Fouriers, human genome, Markov and $k = 5$ central model (chr22); mouseLSTM50 (chr20).

Christian Grønbæk, Yuhu Liang, Desmond Elliott, and Anders Krogh

April 17, 2022

Fouriers plots, predictions on chromosome 22

Markov model

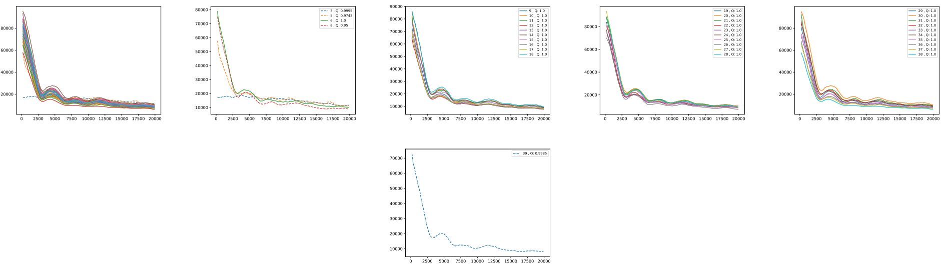

Figure SB1: Markov model, k=14. Fouriers on reference-base probability for chromosome: hg38_chr22. The genome string is divided in adjacent segments of 1Mb; several adjacent segments are covered in each plot, segment numbers are indicated in the legend. First plot (upper left) shows the results for all segments in this chromosome.

$k = 5$ **central model**

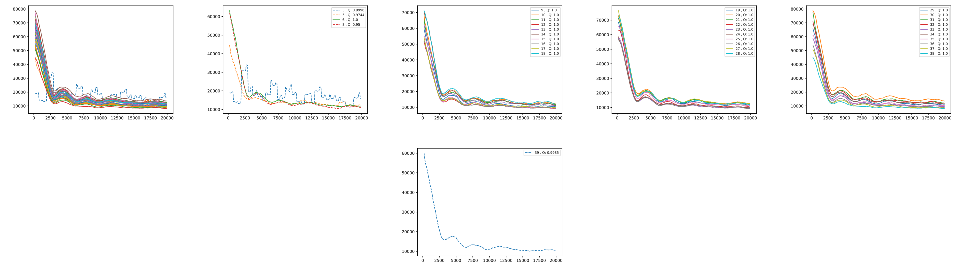

Figure SB2: 5-mer model. Fouriers on reference-base probability for chromosome: hg38_chr22. The genome string is divided in adjacent segments of 1Mb; several adjacent segments are covered in each plot, segment numbers are indicated in the legend. First plot (upper left) shows the results for all segments in this chromosome.

LSTM200

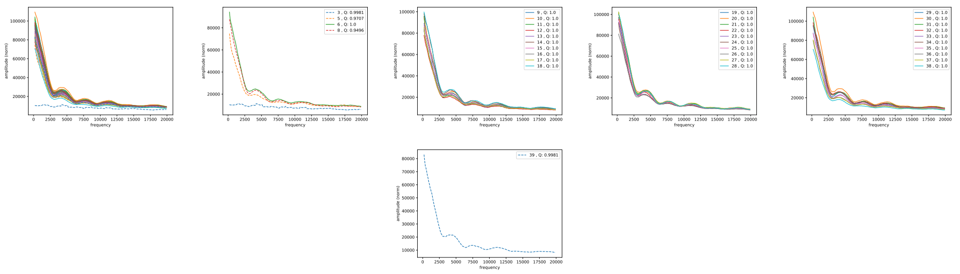

Figure SB3: LSTM200. Fouriers on reference-base probability for chromosome: hg38_chr22. The genome string is divided in adjacent segments of 1Mb; several adjacent segments are covered in each plot, segment numbers are indicated in the legend. First plot (upper left) shows the results for all segments in this chromosome.

Fouriers plots, mouseLSTM50 predictions on chromosome 20

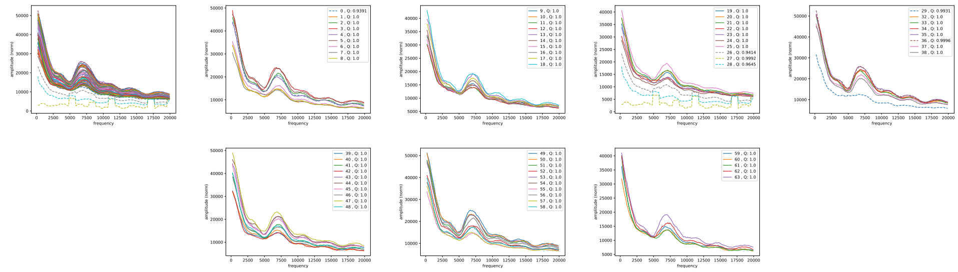

Figure SB4: mouseLSTM50. Fouriers on reference-base probability for chromosome: hg38_chr20. The genome string is divided in adjacent segments of 1Mb; several adjacent segments are covered in each plot, segment numbers are indicated in the legend. First plot (upper left) shows the results for all segments in thise chromosome
