# Supplementary material for: Context dependent prediction in DNA sequence using neural networks
Source: PeerJ. 2022 Sep 20;10:e13666. doi: 10.7717/peerj.13666 (PMC9504454; doi:10.7717/peerj.13666)
Supplement: Supplemental Information 17 [file peerj-10-13666-s017.pdf]

Supplementary IVc: Context dependent prediction in DNA sequence using neural networks. Fourier, zebrafish genome, GC/AT content, low frequencies.

Christian Grønbæk, Yuhu Liang, Desmond Elliott, and Anders Krogh

April 17, 2022

This file contains the first of two sets of plots based on the Fourier analysis showing the L2-norm of the Fourier coefficients in a running window. This first set covers the frequency range from 200 to 45000 using a window length of 1000 (and a step size of 100); the second covers the frequencies from 40000 to 140000 and used a window length of 5000 (and step size of 100) and is placed in a separate file.

Fourier plots, GC/AT content, frequency range 200 to 45000

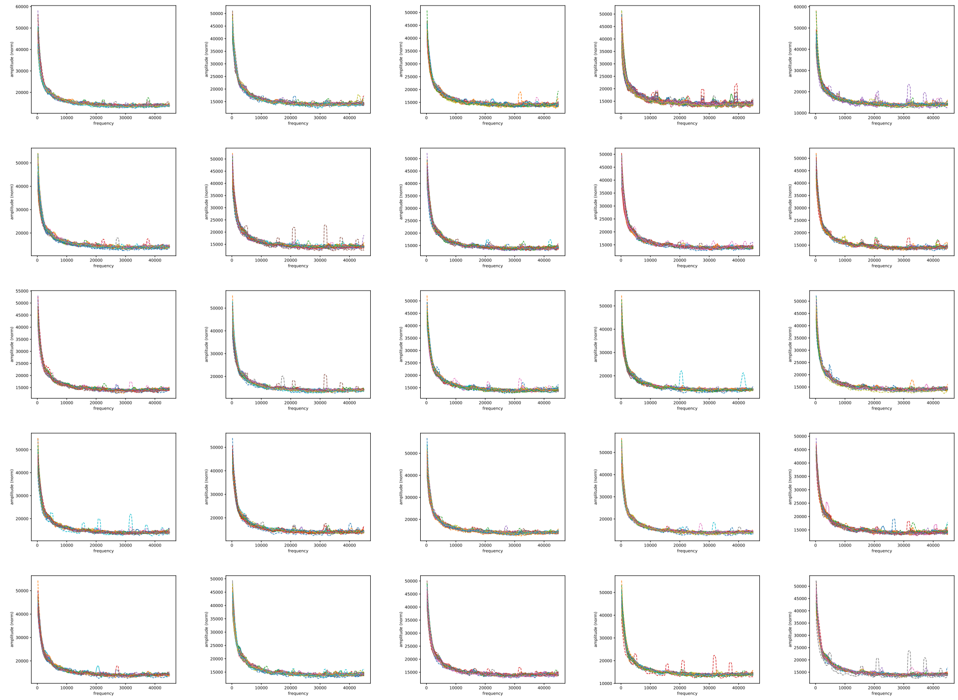

Figure 1: Zebrafish GC/AT content. Fouriers on GC/AT content arrays, low frequency range. Each plot covers one chromosome, listed in increasing order (chr1 to chr25). The genome string is divided in adjacent segments of 1Mb (per chromosome); each plot shows the results for all segments in the chromosome (with ratio of qualified positions > 0.9).
