# Supplementary material for: Context dependent prediction in DNA sequence using neural networks
Source: PeerJ. 2022 Sep 20;10:e13666. doi: 10.7717/peerj.13666 (PMC9504454; doi:10.7717/peerj.13666)
Supplement: Supplemental Information 20 [file peerj-10-13666-s020.pdf]

Supplementary Vb: Context dependent prediction in DNA sequence using neural networks. Fourier, D.melanogaster genome, GC/AT content.

Christian Grønbæk, Yuhu Liang, Desmond Elliott, and Anders Krogh

April 16, 2022

This file contains two sets of plots based on the Fourier analysis showing the L2-norm of the Fourier coefficients in a running window. This first set covers the frequency range from 200 to 45000 using a window length of 1000 (and a step size of 100), while the second covers the frequencies from 40000 to 140000 and used a window length of 5000 (and step size of 100). Note that, since the window length is five times larger in the second set than in the first, the scale of the amplitudes (y-axis) is five times higher in the second set than in the first.

Fourier plots, GC/AT content, frequency range 200 to 45000

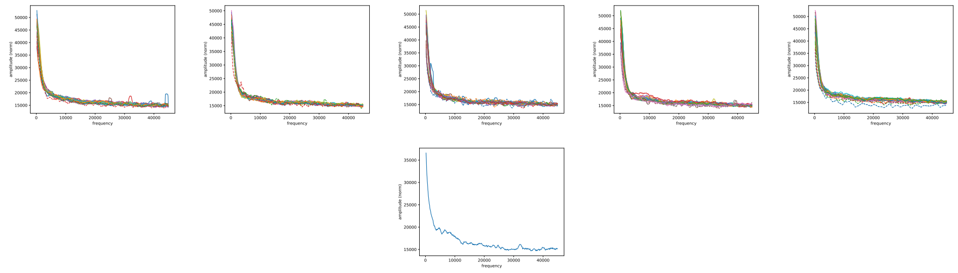

Figure 1: Drosophila GC/AT content. Fouriers on GC/AT content arrays, low frequency range. Each plot covers one chromosome, listed in increasing order (chrX, chr2L, chr2R, chr3L, chr3R, chr4). The genome string is divided in adjacent segments of 1Mb (per chromosome); each plot shows the results for all segments in the chromosome (with ratio of qualified positions > 0.9).

Fouriers plots, GC/AT content, frequency range 40000 to 140000

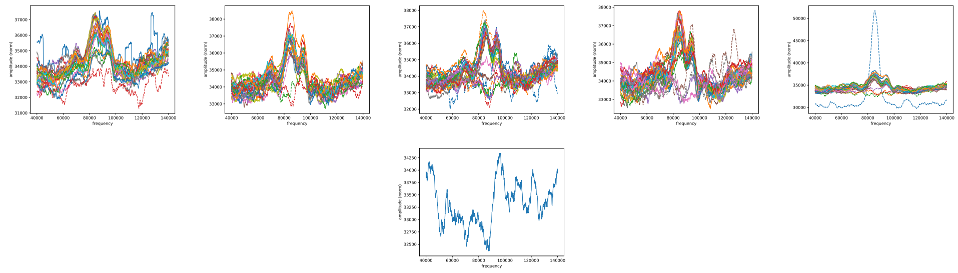

Figure 2: Drosophila GC/AT content. Fouriers on GC/AT content arrays, higher frequency range. Each plot covers one chromosome, listed in increasing order (chrX, chr2L, chr2R, chr3L, chr3R, chr4). The genome string is divided in adjacent segments of 1Mb (per chromosome); each plot shows the results for all segments in the chromosome (with ratio of qualified positions > 0.9).
