# Supplementary material for: Context dependent prediction in DNA sequence using neural networks
Source: PeerJ. 2022 Sep 20;10:e13666. doi: 10.7717/peerj.13666 (PMC9504454; doi:10.7717/peerj.13666)
Supplement: Supplemental Information 22 [file peerj-10-13666-s022.pdf]

Supplementary VIb: Context dependent prediction in DNA sequence using neural networks. Fourier, yeast genome, GC/AT content.

Christian Grønbæk, Yuhu Liang, Desmond Elliott, and Anders Krogh

April 16, 2022

This file contains two sets of plots based on the Fourier analysis showing the L2-norm of the Fourier coefficients in a running window. This first set covers the frequency range from 20 to 4500 using a window length of 100 (and a step size of 10), while the second covers the frequencies from 4000 to 14000 and used a window length of 500 (and step size of 10). Note that, since the window length is five times larger in the second set than in the first, the scale of the amplitudes (y-axis) is five times higher in the second set than in the first.

Fourier plots, GC/AT content, frequency range 20 to 4500

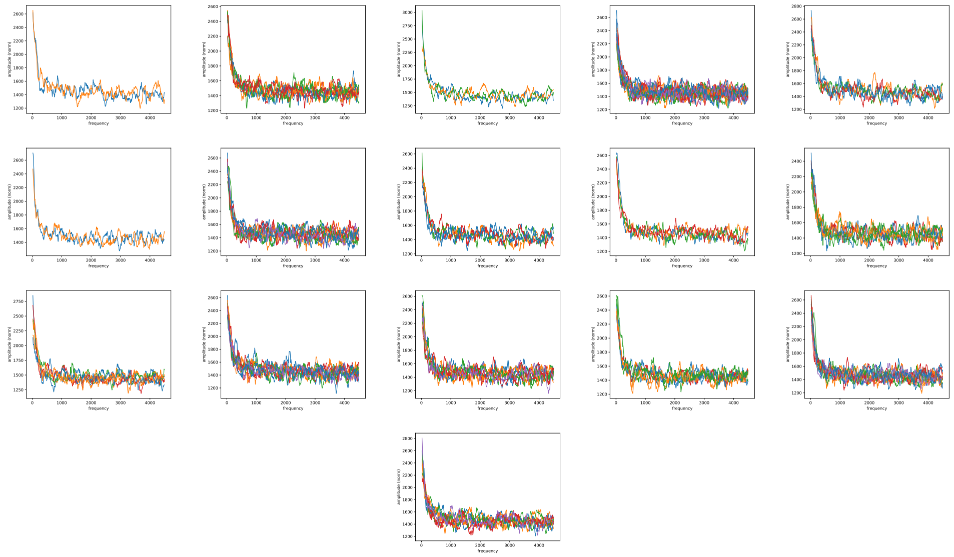

Figure 1: Yeast GC/AT content. Fouriers on GC/AT content arrays, low frequency range. Each plot covers one chromosome, listed in increasing order (chr1 to chr16). The genome string is divided in adjacent segments of 100Kb (per chromosome); each plot shows the results for all segments in the chromosome (with ratio of qualified positions > 0.9).

Fourier plots, GC/AT content, frequency range 4000 to 14000

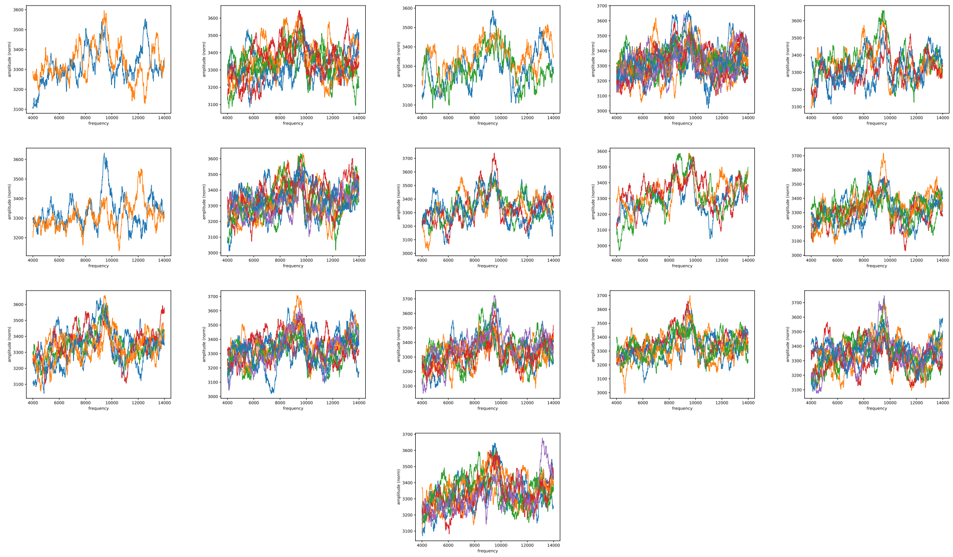

Figure 2: Yeast GC/AT content. Fouriers on GC/AT content arrays, higher frequency range. Each plot covers one chromosome, listed in increasing order (chr1 to chr16). The genome string is divided in adjacent segments of 100Kb (per chromosome); each plot shows the results for all segments in the chromosome (with ratio of qualified positions > 0.9).
